# Molecular Dynamics-Derived Pharmacophore Model Explaining the Nonselective Aspect of K_V_10.1 Pore Blockers

**DOI:** 10.3390/ijms22168999

**Published:** 2021-08-20

**Authors:** Žan Toplak, Franci Merzel, Luis A. Pardo, Lucija Peterlin Mašič, Tihomir Tomašič

**Affiliations:** 1 Faculty of Pharmacy, University of Ljubljana, 1000 Ljubljana, Slovenia; zan.toplak@ffa.uni-lj.si (Ž.T.); lucija.peterlinmasic@ffa.uni-lj.si (L.P.M.); 2 Theory Department, National Institute of Chemistry, 1000 Ljubljana, Slovenia; franci.merzel@ki.si; 3 AG Oncophysiology, Max-Planck Institute for Experimental Medicine, 37075 Göttingen, Germany; pardo@em.mpg.de

**Keywords:** cancer, Eag1, hERG, K_V_10.1 inhibitors, molecular dynamics, pharmacophore

## Abstract

The K_V_10.1 voltage-gated potassium channel is highly expressed in 70% of tumors, and thus represents a promising target for anticancer drug discovery. However, only a few ligands are known to inhibit K_V_10.1, and almost all also inhibit the very similar cardiac hERG channel, which can lead to undesirable side-effects. In the absence of the structure of the K_V_10.1–inhibitor complex, there remains the need for new strategies to identify selective K_V_10.1 inhibitors and to understand the binding modes of the known K_V_10.1 inhibitors. To investigate these binding modes in the central cavity of K_V_10.1, a unique approach was used that allows derivation and analysis of ligand–protein interactions from molecular dynamics trajectories through pharmacophore modeling. The final molecular dynamics-derived structure-based pharmacophore model for the simulated K_V_10.1–ligand complexes describes the necessary pharmacophore features for K_V_10.1 inhibition and is highly similar to the previously reported ligand-based hERG pharmacophore model used to explain the nonselectivity of K_V_10.1 pore blockers. Moreover, analysis of the molecular dynamics trajectories revealed disruption of the π–π network of aromatic residues F359, Y464, and F468 of K_V_10.1, which has been reported to be important for binding of various ligands for both K_V_10.1 and hERG channels. These data indicate that targeting the K_V_10.1 channel pore is also likely to result in undesired hERG inhibition, and other potential binding sites should be explored to develop true K_V_10.1-selective inhibitors as new anticancer agents.

## 1. Introduction

K_V_10.1 (Eag1) is a voltage-gated potassium channel of the ‘ether-à-go-go’ channel family. Within this family, the hERG channel (eag-related gene, K_V_11.1) is known to be responsible for increased risk of malignant cardiac arrhythmia, which can lead to sudden cardiac death [1,2,3]. In contrast, K_V_10.1 is almost not detectable outside the human central nervous system, except in many different tumors, where its expression is dysregulated. The mechanisms by which K_V_10.1 is involved in cancer progression are not yet fully understood, although effects such as increased cancer-cell proliferation, migration, angiogenesis, and resistance to hypoxia have been shown [4,5]. The high expression of K_V_10.1 in 70% of various tumors and cancers make this channel a potential cancer marker and target for anticancer drugs [6].

K_V_10.1 is a homotetramer of four identical subunits, each of which consists of the intracellular N-terminal Per-Arnt-Sim (PAS) domain, and the C-terminal cyclic nucleotide-binding domain and transmembrane portion, which has six α-helical segments (S1-S6). Segments S1 to S4 form the voltage-sensor domain, which is responsible for translating the change in membrane potential into the mechanical action of the pore domain (segments S5, S6) that opens and closes the channel pathway for potassium ions [7]. The cryo-electron microscopy (cryo-EM) structures of K_V_10.1 and hERG have recently been determined in different conformations. The three-dimensional K_V_10.1 structure (PDB: 5K7L [7]) is in the closed state, whereas hERG (PDB: 5VA1 [8]) was defined in the open state. In the open conformation of both of these channels, potassium ions flow through the central cavity and then through the so-called selectivity filter. The selectivity filter forms a narrow pathway that is lined by the carbonyl oxygens of amino acid backbones, and this allows the selective flow of potassium ions, while it is too narrow for the passage of ligands [7]. Therefore, known ligands that bind below the selectivity filter either block the ion flow or modulate the stability of the selectivity filter.

Mutagenesis studies have highlighted the importance of the two aromatic residues Y464 (Y652 in hERG) and F468 (F656 in hERG) in each K_V_10.1 subunit in terms of the binding of various inhibitors. Aromatic residues are important for the formation of π–π stacking interactions between inhibitors and the channel, and for the cation–π interactions with the positively charged groups (usually substituted amines) in many K_V_10.1 and hERG inhibitors. The cryo-EM structure of hERG revealed four cylindrically shaped hydrophobic side pockets that extend from the central cavity toward the bottom of the selectivity filter, with a length of ca. 11 Å and a diameter of 8 Å [8]. The entrances of these pockets in the cryo-EM structure of K_V_10.1 are not accessible due to the closure of the pore domain. On the basis of the high similarity to hERG (sequence similarity and identity of the pore domains: 63%, 51%, respectively [9,10]), it is reasonable to assume that the pockets are reachable in the open K_V_10.1 conformation. A pocket of this size can easily accommodate the substituted aromatic ring of different ligands. Many in silico studies of inhibitors binding to K_V_10.1 and hERG were performed before the cryo-EM structures of the channels were known, which were therefore based on homology models that were created using more distantly related potassium channels [11,12,13]. However, the hydrophobic side pockets were absent in the homology model structures of K_V_10.1 and hERG because none of the channels used as templates had these pockets in their structure. Furthermore, the cryo-EM structures of K_V_10.1 and hERG channels show a negative electrostatic potential in the region directly below the selectivity filter, with many potential hydrogen-bond acceptors that can form the binding site for positively charged moieties [7,8]. 

Hydrophobic and positively charged groups are the most important pharmacophore features of all known three-dimensional (3D) pharmacophore models of the hERG channel that have been constructed, although no such models have been generated for K_V_10.1 inhibitors [14,15,16]. The reason for this is the great interest in blocking the cardiac hERG channels in drug development, as hERG inhibition is an undesirable side-effect that results in safety concerns. In addition, the number of known K_V_10.1 inhibitors is very small, but a better understanding of K_V_10.1 inhibition and binding of known ligands might lead to improved design strategies and the development of new anticancer drugs that act as K_V_10.1 inhibitors [17].

In the present study, we combined the use of homology modeling, molecular docking, molecular dynamics (MD) simulations, and pharmacophore modeling, which have not been used together before, to study the binding of voltage-gated potassium channel modulators. The study of the binding of nonselective K_V_10.1 inhibitors into the channel pore will help us to reveal the common structural features that are involved in K_V_10.1 and hERG inhibition, and to develop improved strategies for the design of selective K_V_10.1 inhibitors. We used the open hERG structure to model K_V_10.1 in the open pore domain state that is required for inhibitor binding. Next, we combined docking and MD simulations to determine the binding of ligands to the K_V_10.1 homology model, followed by the generation of structure-based pharmacophore models based on the movement of ligands across the MD trajectory. The dynamic pharmacophore models generated were examined to study the interactions of the ligands in the binding site below the selectivity filter. The MD pharmacophore models for different ligands were then merged to build a general structure-based pharmacophore model for K_V_10.1 inhibitors. The model was then validated by screening other known K_V_10.1 channel inhibitors. Furthermore, the final pharmacophore model was compared with models that were previously generated for hERG channel inhibitors [15,18]. Our new methodology thus incorporates multiple molecular modeling techniques, and it has allowed us for the first time to create a structure-based pharmacophore model for K_V_10.1 inhibitors that can be used to rationalize the structure–activity relationships observed, to identify novel K_V_10.1 inhibitors by virtual screening, and to design novel ligands that have anticancer activities through their inhibition of K_V_10.1.

## 2. Materials and Methods

### 2.1. Software

For homology modeling, the T-coffee web server [19] was used for the initial sequence alignment, and MODELLER 9.21 [20] for the model construction. VERIFY 3D [21], ERRAT [22], PROVE [23], and PROCHECK [24] were used for model validation. The initial docking experiments were performed using Glide in the Schrödinger Drug Discovery Suite (v2018-1) [25]. MD simulation systems were prepared using the Membrane builder [26] input generator module of the CHARMM-GUI online server [27]. NAMD (version 2.9) [28] and CHARMM36 [29] force field were used for the MD simulations. LigandScout 4.4 Expert (Inte:Ligand GmbH., Maria Enzersdorf, Austria [18,30]) was used for analysis of the ligand interactions in the MD simulation, and for the generation of the pharmacophore models and the virtual screening for model validation. The library for the pharmacophore model validation was prepared using the CHEMBL dataset [31] for hERG compounds, then filtered and processed using the KNIME Analytics Platform [32], with the addition of OpenBabel [33], RDKit [34], and Inte:Ligand Expert KNIME Extensions nodes [35].

### 2.2. Homology Modeling

The sequence of human K_V_10.1 was downloaded from Uniprot [36] (O95259) and aligned with the sequence of the hERG channel from the PDB structure (5VA1 [8]), using the Expresso algorithm from the T-Coffee web servers [19]. The sequence was then visually inspected and further modified to match previously published data [9]. MODELLER 9.21 [20] was used to generate 100 homology models using the hERG channel (PDB 5VA1) as template. The unresolved loops and side chains were constructed by MODELLER [20], with modified parameters for the C_α_ symmetry between all four of the subunits, and α-helix constraint for portions of the voltage-sensor domain with lower resolution in the hERG cryo-EM structure, compared to the K_V_10.1 cryo-EM structure.

### 2.3. Homology Model Evaluation

Homology model evaluation was performed for the 10 best and 10 worst models created. They were selected based on the combination of the scoring functions molpdf, Discrete Optimized Protein Energy (DOPE), and GA341, as computed by MODELLER 9.21 [20]. Geometric errors were calculated using VERIFY 3D [21], ERRAT [22], PROVE [23], and PROCHECK, which include phi-psi outliers, overall model quality, secondary structure evaluation, and deviation from standard atomic volumes [24]. 

### 2.4. Docking of Compounds

**Figure 1:**
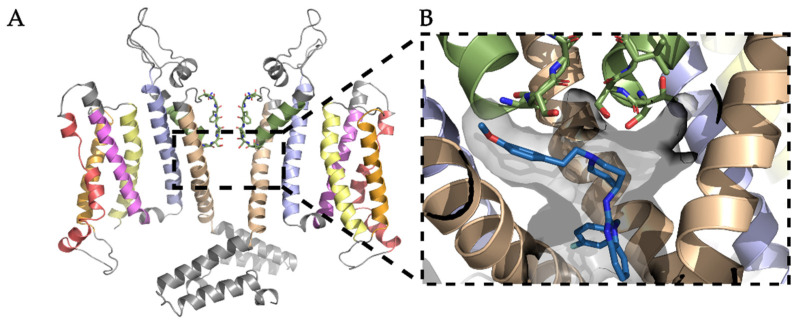
(**A**) Best homology model of the transmembrane part of the K_V_10.1 channel in the open conformation, with only two opposing subunits shown for better clarity. The subunits are colored by domain: S1, yellow; S2, orange; S3, red; S4, purple; S5, blue; S6, brown; pore, green; rest of protein not embedded in the membrane, gray. The selectivity filter in the pore domain is shown as green sticks. (**B**) Enlarged view from (**A**) (as indicated) to show the central cavity that is the binding site for various K_V_10.1 pore blockers, with only three subunits shown for clarity. The binding site is shown as a gray surface, with astemizole in blue sticks, oriented into one of the hydrophobic side pockets below the selectivity filter.

Ligand docking was performed using Schrödinger Drug Discovery Suite (version 2018-3) [25]. The homology model prepared (Figure 1) was used for grid generation in the Schrödinger Maestro software. The box in Figure 1 that represents a region for a potential binding site is positioned below the selectivity filter, without defining any further constraints. Ligands (Table 1) were protonated using OpenBabel (version 2.4.0), with pK_a_ set to 7.4 [33]. Conformations were generated using the Schrödinger Suite ligand software LigPrep, with a maximum of 1000 conformations per ligand, and with the other settings set to their default values. For docking in Schrödinger Suite, the Glide SP protocol was chosen with the poses per ligand set to 100.

### 2.5. Molecular Dynamics Preparation and Simulation

The NAMD [28] simulation package (version 2.9) and the CHARMM36 [29] force field were used for the MD simulation. The corresponding parameters for the ligands were derived from geometry-optimized structures using the suite of programs in Gaussian16 [42] and the PARAMCHEM CGENFF [43,44] website. Three potassium ions were manually placed in the selectivity filter of the channel at the S0, S2, and S4 binding sites of selectivity filter with two water molecules in between, as published previously [45]. The ligand–protein complex was embedded in a 140 × 140 Å phosphatidylcholine (POPC) lipid bilayer, and solvated with transferable intermolecular potential 3P (TIP3P) water molecules, and the system was neutralized by setting the 0.15 M KCl solution with Charmm-GUI. Each of the systems contained about 250,000 atoms, and they were all initially minimized using the steepest descent method for 500 steps, followed by 1000 steps of the adaptive Newton–Raphson method; they were then heated and equilibrated at 300 K for 5 ns. The systems were parameterized using the CHARMM36 force field. Production runs of 100 ns per system were performed under the isothermal-isobaric (NPT) conditions. Temperature and pressure were controlled using a Nose-Hoover thermostat and piston [46]. Long-range electrostatics were calculated using the particle mesh Ewald (PME [47]) method with a 12 Å cut-off, with switching and pair list distances of 10 Å and 16 Å, respectively. All of the chemical bonds between hydrogen and heavy atoms were held fixed using the SHAKE algorithm [48], while an integration time step of 2 fs was used.

### 2.6. Analysis of Molecular Dynamics Simulation

The protein stability was assessed by root mean square fluctuation (RMSF) analysis per residue using Python library MDAnalysis [49,50]. The protein and ligand were first aligned to the first frame using VMD [51] root-mean-square deviation (RMSD) Trajectory Tool, and 5000 uniformly distributed conformations were extracted. The aligned trajectory was analyzed using Python library MDAnalysis, and the RMSF values for all of the systems were plotted using Python library Matplotlib. Pairwise analysis was performed instead of classical RMSD calculations of ligand stability, using Python library MDAnalysis, to better represent the changes in the ligand RMSD as the simulation progressed, rather than just for the first conformation at the beginning of the simulation. The ligand from 5000 uniformly distributed conformations was extracted from the full trajectory, and the analyzed data were plotted using Python library Matplotlib.

### 2.7. Pharmacophore Modeling

For the MD trajectory analysis using LigandScout 4.4 Expert, all of the MD trajectories of the protein–ligand complexes were preprocessed to contain 500 evenly distributed frames across the entire production run. The MD trajectories were used to generate ensembles of structure-based pharmacophore models for each complex, to analyze the ligand–protein interactions. From the last 20 ns of each MD simulation, the four most frequently occurring pharmacophore models in the MD trajectory were selected, and these were merged to generate the merged pharmacophore model for each ligand–protein complex. These merged pharmacophore models for each ligand-protein complex were further merged into a model that represented the final merged K_V_10.1 structure-based pharmacophore model, which describes the important pharmacophore features of all of the simulated K_V_10.1 inhibitors. Based on the individual merged structure-based pharmacophore models and validation of the models by virtual screening, the features of the final merged K_V_10.1 model were manually adjusted to increase the performance.

### 2.8. Virtual Library Preparation

Validation of the final merged K_V_10.1 structure-based pharmacophore model was performed by screening against two libraries. The first library contained 15 compounds with known IC_50_ values for K_V_10.1 inhibition and a known binding site in the channel pore (Supporting Information Table S1) [17]. The second library contained compounds (‘decoys’) that are not likely to inhibit K_V_10.1. These decoys were generated using two approaches. The first set of decoys was generated using the Database of Useful Decoys: Enhanced (DUDE) decoy online server [52]. For each of the 15 known K_V_10.1 inhibitors, the DUDE server generated 50 decoy molecules, which resulted in 750 decoy ligands. Compared to known inhibitors, the decoys generated had similar 1D physicochemical properties, but dissimilar 2D topologies. The second decoy set was generated using compounds that lacked hERG inhibitory activity, as there are no reported inactive compounds for K_V_10.1 in the literature. Inactive compounds for the hERG channel were selected based on the high similarity of the binding sites between these two channels. The compounds were retrieved from the ChEMBL database [31] using Target CHEMBL ID (CHEMBL240) for hERG using the KNIME Analytics Platform [32]. Compounds with declared IC_50_ ≥ 100 μM were selected as inactive. In addition, the selected compounds were filtered to remove duplicates and protonated (pH 7.4) using the additional OpenBabel [33], RDKit [34] nodes in the KNIME workflow. The second decoy library contained 448 decoy ligands (Supporting Information Table S2). The active and decoy libraries in the SMILES format were transformed into multiconformational LigandScout libraries (.ldb) using the algorithms of LigandScout with default setting “BEST” (maximum number of conformers per molecules, 200; timeout: 600 s; RMS threshold, 0.5; energy window, 15.0; maximum pool size, 4000; maximum fragment build time, 30 s).

### 2.9. Virtual Screening

To validate the final merged K_V_10.1 structure-based pharmacophore model in LigandScout, virtual screening was performed to refine the model and to select the one that best discriminated between the active and decoy molecules. The settings used in LigandScout screening were: scoring function, pharmacophore-fit; screening mode; match all query features; retrieval mode, stop after first matching conformation; maximum number of omitted features, 0; check exclusion volumes, true. 

## 3. Results and Discussion

Unfortunately, all of the known K_V_10.1 inhibitors also inhibit the highly similar cardiac hERG channel, and therefore these have limited potential for development into anticancer drugs that act through this novel mechanism of action. With the goal being to develop selective K_V_10.1 inhibitors as potential anticancer drugs [53], we investigated their binding modes in the K_V_10.1 channel pore using advanced molecular modeling methodologies. The creation of the common structure-based pharmacophore model for K_V_10.1 inhibitors binding to the channel pore allowed us to compare it with the known hERG ligand-based pharmacophore models and to assess the potential for targeting the K_V_10.1 channel pore for the development of K_V_10.1-selective anticancer agents.

### 3.1. Homology Modeling of the K_V_10.1 Open Pore Conformation

**Figure 2:**
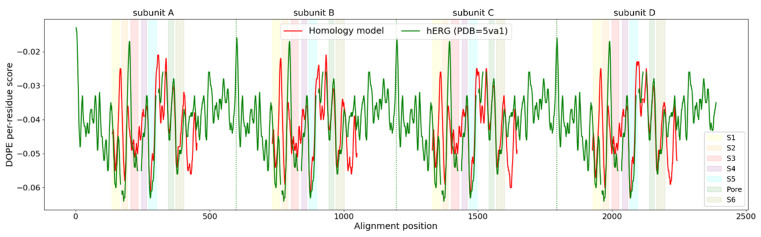
Evaluation of the selected homology model using MODELLER 9.21 DOPE scoring function. Green line, (PDB: 5VA1) DOPE per residue score for hERG used as template; red line, DOPE per residue score of the K_V_10.1 homology model created and used in this study. The different color zones represent the S1 to S6 transmembrane segments and the pore domain: S1, yellow; S2, orange; S3, red; S4, purple; S5, blue; S6, brown; pore domain, green. Vertical green dotted lines divide subunits A, B, C, and D. High root mean square fluctuation values in the white zones represent amino acids that are not in the transmembrane part, but in the intracellular and extracellular loops.

**Figure 3:**
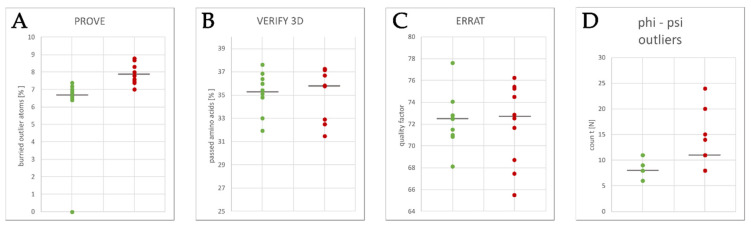
Geometric errors report for the 10 best (green dots) and 10 worst (red dots) homology models generated by the model evaluation software (as indicated). Significant differences were observed only in the PROVE calculation of the buried outlier atoms. Model evaluation was done using software (**A**) PROVE to calculate the buried outlier atoms, (**B**) VERIFY 3D to evaluate secondary structure of the model, (**C**) ERRAT to verify model structure based on the statistics of non-bonded atom-atom interactions and, (**D**) PROCHECK to calculate geometry of amino acids to find phi-psi outliers.

As pore blockers bind to the open pore conformation of K_V_10.1, and the cryo-EM of rat K_V_10.1 was solved in the closed pore conformation, we first built a homology model of the open pore conformation of K_V_10.1 based on the hERG structure as template (Figure S1 in Supplementary Materials) [7,8]. The hERG channel was selected based on the 63% similarity in the pore domain with K_V_10.1 [9]. The homology model of the open pore conformation of K_V_10.1 for further experiments (Figure 1, Figure S1) was selected based on the combination of the scoring functions (Figure 2) and evaluation of geometric errors calculated using VERIFY 3D [21], ERRAT [22], PROVE [23], and PROCHECK, which include phi-psi outliers, overall model quality, secondary structure evaluation, and deviation from standard atomic volumes (Figure 3). There were only small variations at the beginning and the end of each subunit, which are of minor importance, as the binding site is not located in that part of the protein (Figure 2). The only statistically significant difference was in the PROVE calculation of buried outlier atoms, which was in favor of the best models. The best model for docking was selected based on the evaluation results and visual inspection. On visual inspection, models that had extracellular loops above the selectivity filter or in any other unusual conformation were removed.

### 3.2. Docking of K_V_10.1 Inhibitors for Binding to the Channel Pore

**Figure 4:**
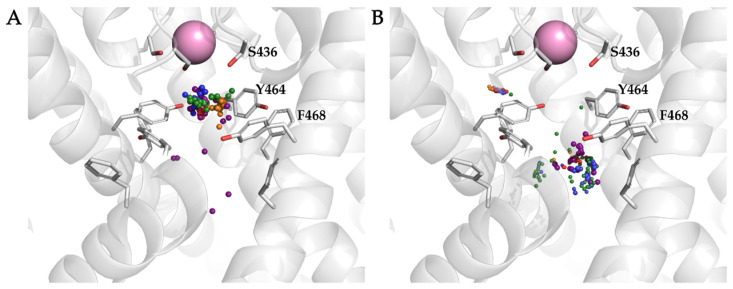
Distribution within the homology model of the K_V_10.1 open pore conformation of amines (**A**) and aromatic moieties (**B**) in the top 20 ranked poses per ligand. The small spheres in different colors represent nitrogen amine atoms (**A**) and geometric centers of aromatic rings (**B**) of the docked ligands. Spheres that represent astemizole, clofilium, imipramine, MK-499, and quinidine are blue, orange, green, red, and purple, respectively.

All of the compounds were docked to the central cavity of the K_V_10.1 channel of the homology model of the K_V_10.1 open pore conformation using Schrödinger’s Glide docking software. As the pore is symmetric, there are four possible orientations for each ligand in the putative binding site below the selectivity filter (Figure 1B). The 100 highest-ranked docking poses per the ligands analyzed and the binding of the ligands to only one of these four sites was considered in the further analysis. The calculated binding affinities of the docked ligands (i.e., GlideScore values) were in relatively good agreement with the experimental data (Table 2). One of the outliers was clofilium, which ranked in the group of MK-499 and imipramine with an affinity of ~30 μM, although clofilium is one of the most potent K_V_10.1 inhibitors. The amines and aromatic rings of the top-ranked 20 docking poses per ligand were analyzed. The location of each amine (colored spheres in Figure 4A) and aromatic ring (colored spheres in Figure 4B) are visualized in Figure 4. Almost all of the amines were located in close proximity to S436 of K_V_10.1 (Figure 4A), directly below the entrance to the selectivity filter of the channel, and in the same plane as the aromatic residue Y464. S436 and Y464 are described in the literature as important residues for binding and stabilization of the ligands [41,54]. Aromatic rings of studied ligands were in close proximity to F468 (Figure 4B), making their position suitable for π-stacking interaction.

**Figure 5:**
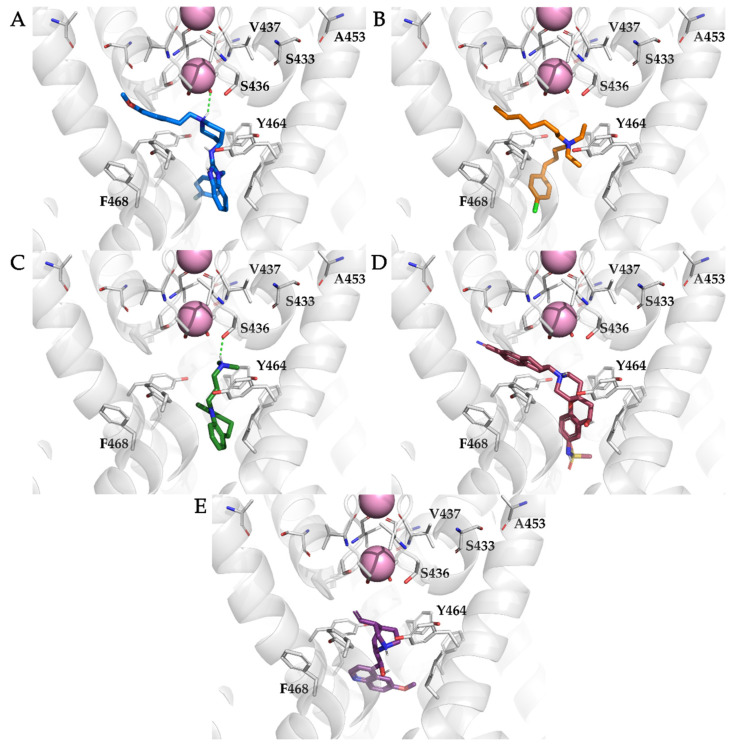
Docking poses within the homology model of the K_V_10.1 open pore conformation of astemizole ((**A**); blue sticks), clofilium ((**B**); orange sticks), imipramine ((**C**); green sticks), MK-499 ((**D**); pink sticks), and quinidine ((**E**); magenta sticks) selected for molecular dynamics simulations. The K_V_10.1 amino-acid residues reported in the literature as important for the binding of the ligands used in this study are labeled and shown as gray sticks. Potassium ions are shown as pink spheres. Hydrogen bonds are shown as green dashed lines.

The binding pose of each ligand for the MD simulation of the homology model of the K_V_10.1 open pore conformation was selected from the top 20 ranked poses based on the Schrödinger GlideScore scoring function score (Figure 5). The binding poses did not differ significantly from each other, except for clofilium and quinidine (Figure S2), where Glide scored the horizontally mirrored poses similarly. For clofilium, a pose with an aromatic ring in the lower part of the central cavity was chosen, which was similar to that reported for the hERG channel, because of the proximity of the aromatic rings of Y464 and F468, which have a strong influence on clofilium binding to hERG (Figure 5B) [55,56]. Quinidine was docked with a quinuclidine moiety (an aliphatic moiety with amine) that pointed either toward the selectivity filter or in the opposite direction, toward the exit of the central cavity. The highest scoring pose of quinidine with the quinuclidine moiety below the selectivity filter was selected for MD simulation (Figure 5E). The selected pose placed the cationic center below the selectivity filter, where there is negative electrostatic potential that also correlates with the placement of the cationic centers of other ligands [8].

### 3.3. Molecular Dynamics Analysis of Ligand and Protein Stabilities

**Figure 6:**
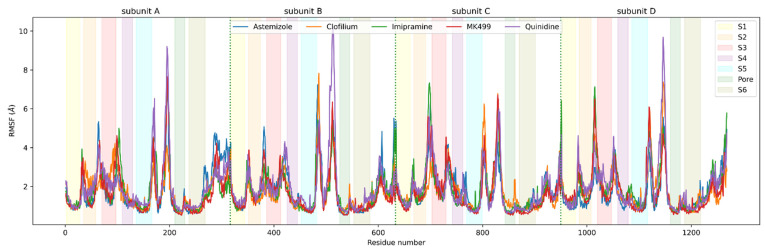
Root mean square fluctuation (RMSF) values of the individual amino-acid residues of the homology model of the K_V_10.1 open pore conformation for each 100th frame in the molecular dynamics trajectories for all of the systems. The different color zones represent the transmembrane segments S1-S6 and the pore domain: S1, yellow; S2, orange; S3, red; S4, purple; S5, blue; S6, brown; pore domain, green. The vertical green dotted lines separate subunits A, B, C, and D. High RMSF values in the white zones represent amino acids that are not embedded in the membrane.

We examined the stabilities of the K_V_10.1 structures (as segments S1–S6) in these MD simulations of the protein–ligand complexes to determine any differences between them. The RMSF values of the C_α_ atoms of all of the amino acid residues were calculated to evaluate the flexibility of each residue (Figure 6). As expected, the parts of the protein that were not embedded in the lipid bilayer (Figure 6, white areas that represent intracellular and extracellular loops; Figure 1A) had higher RMSF values due to the greater flexibility of the amino acids in the water environment than the membrane (Figure 6, colored bars representing transmembrane segments S1–S6; Figure 1A), which had RMSF values < 2 Å. In general, there were no important differences in the RMSF values between the protein–ligand complexes.

**Figure 7:**
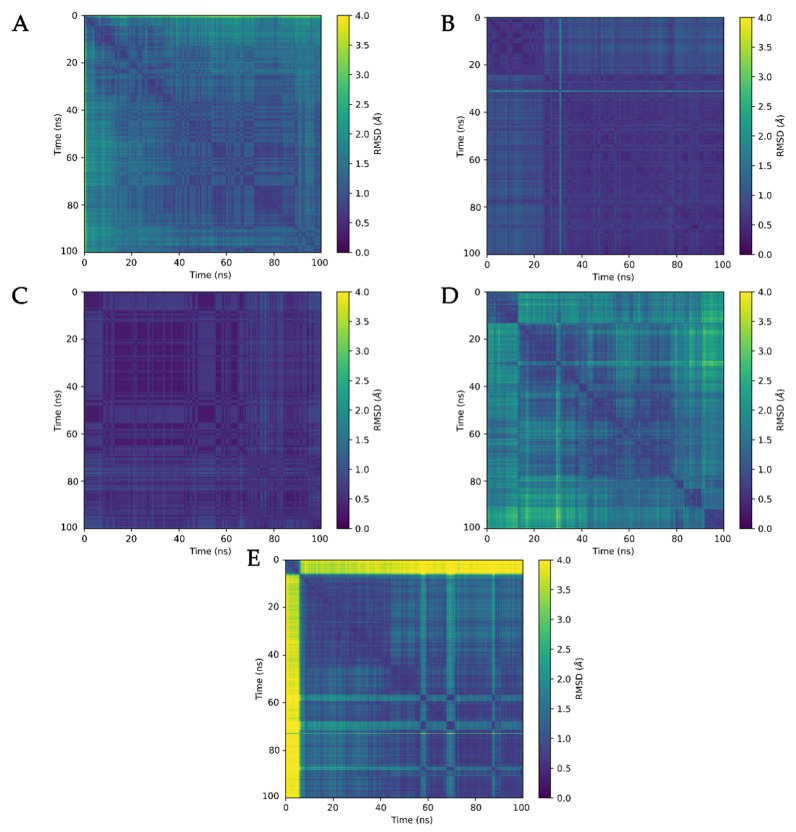
Ligand stabilities in MD simulations analyzed using pairwise RMSD calculations, for astemizole (**A**), clofilium (**B**), imipramine (**C**), MK-499 (**D**), and quinidine (**E**). Large color differences next to the diagonal line indicate major conformational changes compared to the neighboring frames. A representative example can be seen for (**E**), where quinidine was closer to the selectivity filter, and the quinolone moiety was rotated to form π–π interactions with F468 (see main text).

We also analyzed the ligand stabilities using pairwise RMSD analysis performed with MDAnalysis Python library (Figure 7). Pairwise analysis was used to better understand the changes in the ligand binding modes during the simulations, as the RMSD values of one frame can be compared to those of any other frame in the simulation. The binding modes of all of the simulated ligands in the pore of K_V_10.1 stabilized after the first 20 ns, as reflected by the RMSD values of <2 Å. The most stable ligands were clofilium and imipramine, while astemizole, quinidine, and MK-499 showed small conformational changes, although without significant change in the binding mode after the first 20 ns of the production run. The changes in ligand conformation can be seen in Figure 7 as horizontal and vertical yellow bands. For example, quinidine has shifted significantly in the first 8 ns of the simulation as it approaches the selectivity filter, indicated by the yellow color representing high RMSD values (Figure 7E). Smaller shifts of the ligand are shown as less noticeable color changes, as in the case of clofilium, especially after 30 ns of the simulation (Figure 7B).

### 3.4. Analysis of Binding Interactions of K_V_10.1 Inhibitors in the Molecular Dynamics Simulations

**Figure 8:**
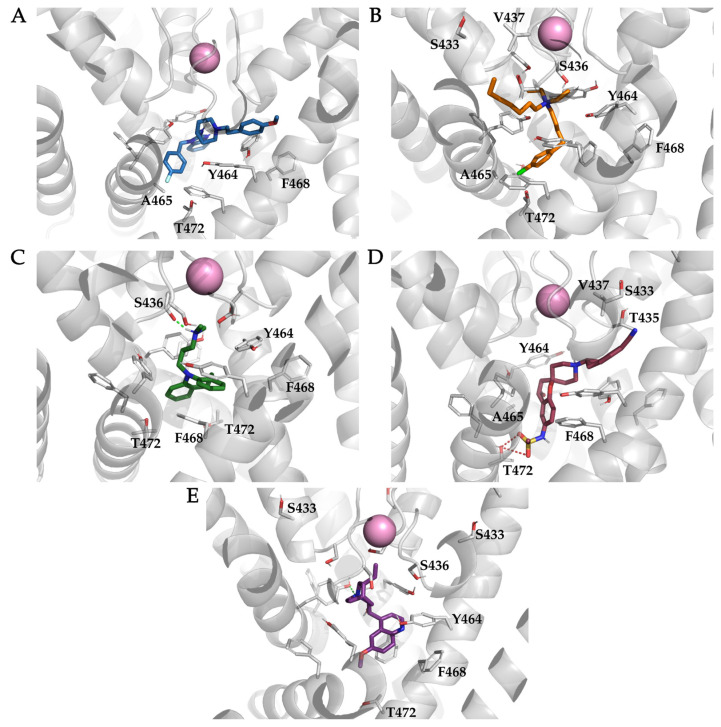
Stable binding poses within the homology model of the K_V_10.1 open pore conformation for the ligands astemizole ((**A**); blue sticks), clofilium ((**B**); orange sticks), imipramine ((**C**); green sticks), MK-499 ((**D**); pink sticks), and quinidine ((**E**); magenta sticks) from the last 20 ns of the simulations. The K_V_10.1 amino-acid residues reported in the literature as important for the binding of the ligands used in this study are labeled and shown as gray sticks. Potassium ions are shown as pink spheres. Hydrogen bonds are shown as green (hydrogen bond donors) and red dashed lines (hydrogen bond acceptors).

LigandScout [30] pharmacophore feature occurrence was used to analyze the interactions between the ligands and the K_V_10.1 channel (Table 3, Figure S3). A set of 500 structure-based pharmacophore models per K_V_10.1–ligand complex was generated using the MD analysis tools in LigandScout. Figure S3 shows the plots of the unique and most frequently appearing models, the total number of interaction features they contain (*x*-axis), and the frequency (number of appearances; *y*-axis) at which they occurred during the last 20 ns of the simulation. Astemizole, clofilium, and MK-499 extended into the eag-family specific hydrophobic side pockets below the selectivity filter (Figure 1B and Figure S4). Residues S433, T435, S436, V437, and A453 have been reported to have important effects on the binding of various ligands and are located in the hydrophobic side pockets. In the K_V_10.1 channel, the A453S mutation reduces the inhibitory activity of clofilium, MK-499, and quinidine [41]. Tertiary amine analogs of clofilium showed approximately 2-fold greater inhibition of hERG when the S641A mutation (A453 in K_V_10.1) was present, which is located downstream of the selectivity filter and upstream of the hydrophobic side pocket [56]. In the simulation of K_V_10.1 here, A453 is too far from the binding position of clofilium, MK-499, and quinidine to come into contact with them during the simulation. Therefore, it is likely that the structural changes in the A453S mutant involve the neighboring residues, and possibly alter the hydrophobic side pockets of K_V_10.1 (Figure 8). Interestingly, A453S has a smaller effect on the inhibition of K_V_10.1 by quinidine (two-fold increase in IC_50_) than on that of clofilium (eight-fold increase in IC_50_). These experimental observations are consistent with our docking experiments and the MD simulations, where quinidine does not bind in the hydrophobic side pockets of K_V_10.1. However, only a three-fold increase in IC_50_ was recorded for MK-499, which in the present simulation enters the hydrophobic side pocket to a similar extent as clofilium [41]. There were no interactions with A453 and the anisole ring of astemizole, which binds similarly to the benzonitril moiety of MK-499. Although astemizole is a well-studied K_V_10.1 and hERG inhibitor, mutational studies with residues deeper in the hydrophobic side pockets are still lacking.

Here, S433 is in close proximity to A453, on the opposite α-helix located in the pore segment. Mutation of S433 to alanine reduces clofilium inhibition of K_V_10.1 by a factor of two, whereas mutation to cysteine has almost no effects. In these MD simulations, we did not observe any interactions between S433 and clofilium (Figure 8B), although there was formation of a short-lived hydrogen bond with the benzonitrile of MK-499 (Figure 8D). Interestingly, mutations S433A and S433C decrease the IC_50_ values of inhibition of K_V_10.1 by quinidine, even though S433 is located too deep in the hydrophobic side pocket to form any interactions with quinidine (Figure 8E); quinidine remained below the entry into the selectivity filter throughout the simulation [41]. Additional structural studies on the effects of the S433A and S433C mutations on the channel structure and the binding of different ligands are needed to better understand the importance of this residue for inhibitor binding.

There is a serine residue in the lower part of the hydrophobic side pocket at the entrance to the selectivity filter in the K_V_10.1 and hERG channels (S436 in K_V_10.1; S624 in hERG). Compared to the wild-type K_V_10.1 channel, the S436T mutation reduces the inhibition of K_V_10.1 by clofilium by a factor of ~10, whereas in combination with the V437I mutation, the inhibition is reduced by a factor of almost 40 [41]. The same mutations have much weaker effects on quinidine inhibition of K_V_10.1, with 2-fold and 3-fold increases in IC_50_, respectively. In the hERG channel, the S624 mutation reduces inhibition by clofilium, whereas residues T623 (T435 in K_V_10.1) and V625 (V437 in K_V_10.1) that are adjacent to S624 are also important for binding [57]. A mutation study with V625A also showed a reduction by a factor of 4 in hERG inhibition by quinidine, which is consistent with the effects of the K_V_10.1 V437 mutations [58]. For clofilium, there were hydrophobic interactions with residues T435 and V437 for 93% and 90% of the simulation time, respectively, while interactions with S436 were not detected (Figure 8B, Table 3). Interactions formed in the hydrophobic side pocket of K_V_10.1 by the aliphatic tail of clofilium were present throughout the simulation. The importance of these interactions might explain why clofilium analogs with shorter aliphatic tails show reduced inhibition of the hERG channel [59]. Hydrophobic interactions with T435 and V437 were present in more than 90% of the simulations with astemizole, and they were also present in the simulations with MK-499 at frequencies of 48% and 80%, respectively (Figure 8D, Table 3). The valine mutation to alanine in hERG (V625A), which corresponds to V437A in K_V_10.1, reduced MK-499 inhibition by a factor of 50, which indicated the great importance of this residue for MK-499 binding. Similarly, the mutation to alanine of the neighboring hERG residues T623 and S624 showed reduced MK-499 inhibition, although to a lesser extent than the V625 mutation [54]. The T435 and V437 residues that are conserved in K_V_10.1 interacted with MK-499 in this MD simulation, which might therefore confirm the binding of MK-499 in the hydrophobic side pocket of K_V_10.1 (Figure 8D). Imipramine showed the most pronounced interactions with S436 in these simulations, with more than 95% occurrence of hydrogen bonding between the imipramine amine and the hydroxyl group of S436 (Table 3). However, there are no data available on the effects of the S436 mutation on imipramine inhibition of K_V_10.1.

Cationic centers are well-known features of hERG inhibitors, and these are present in most of the previously created ligand-based pharmacophore models [14,15,16]. Cation–π interactions were most prominent for MK-499 (61% occurrence) and astemizole (19% occurrence) (Table 3). The amines that represent these cationic centers can also form hydrogen bonds, e.g., with S436 at the entrance of selectivity filter, although these interactions were mainly formed with imipramine (Figure 7C and Figure 8C). We assume that the cationic centers of the ligands are located below the selectivity filter, due to the negative electrostatic potential located there [8]. Comparing the initial docking binding poses of the ligands with the binding poses at the end of the simulations, the cationic centers of all of the ligands were seen to have moved closer to the entrance of the selectivity filter. For astemizole, clofilium, and MK-499, which entered the hydrophobic side pocket, the movement of the cationic center also moved the ligands deeper into the side pocket (i.e., ~2.5 Å for astemizole, ~2.0 Å for clofilium, ~2.4 Å for MK-499). 

**Figure 9:**
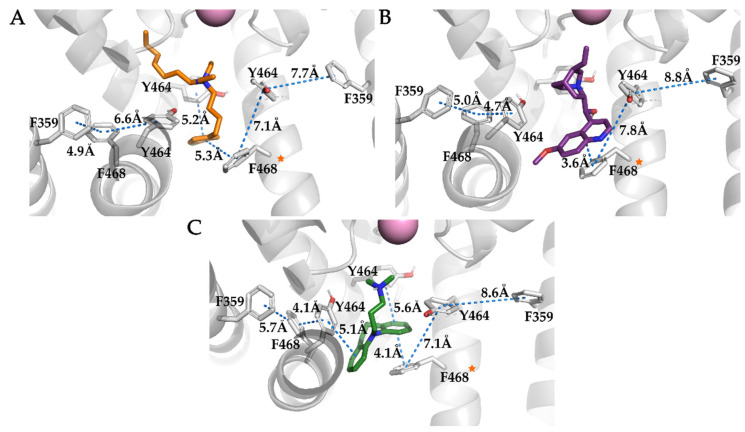
Rotation of the F468 side chain (orange star) upon ligand binding within the homology model of the K_V_10.1. The distances measured between the aromatic network formed with residues F359, Y464, F468, and the ligands are shown with blue dashed lines. Uncoupling between F359 and Y464 of the subunit with rotated F468 shows ~2–3 Å increase in distance between the aromatic rings. One subunit is hidden to increase the visibility of the interactions. (**A**) Clofilium (orange sticks) forms T-shaped and parallel-displaced π–π interactions. (**B**) Imipramine (green sticks) forms two T-shaped and one parallel-displaced π–π interaction. (**C**) Quinidine (purple sticks) forms a parallel-displaced π–π interaction.

The most studied residues in K_V_10.1 and hERG for ligand binding are the aromatic tyrosine (Y464 in K_V_10.1; Y652 in hERG) and phenylalanine (F468 in K_V_10.1; F656 in hERG) residues in the central cavity (Figure 8). Mutations Y652A and F656A in hERG increased the IC_50_ for clofilium by 1329-fold and 484-fold, respectively [56]. In the present simulation, the aromatic ring of clofilium formed hydrophobic interactions with aromatic residues Y464 and F468 throughout the simulation, and based on the LigandScout [30] analysis, 5% of these were π–π interactions (Figure 8B). Visual inspection of the MD trajectory revealed T-shaped π–stacking of Y464 and the aromatic ring of clofilium (Figure 9A). These interactions were formed with Y464 and F468 from the adjacent subunits. The α-helix of segment S6 was slightly rotated, such that the side chain of F468 was oriented toward the channel pore (lower part of the central cavity), directly below the aromatic ring of clofilium. Although LigandScout [30] detected only a brief occurrence of π–π interactions between clofilium and Y464/F468, there was some interesting stacking of the aromatic rings of the neighboring subunits and clofilium in the molecular trajectory. The aromatic rings of Y464 and clofilium resembled T-shaped π–stacking, and F468 and the chlorophenyl moiety in parallel displaced π–stacking orientation at a distance of ~5 Å (Figure 9A). A similar rotation of the side chain of F468 in the central cavity was also seen in the simulations of imipramine and quinidine (Figure 9B,C). The chlorophenyl moiety of clofilium formed several halogen bonds with residues Y464, A465, and T472, as well as hydrophobic interactions similar to those of the fluorophenyl moiety of astemizole. Among the other interactions of the quaternary amine, several of the π–stacking interactions and potential halogen bonds of the chlorophenyl moiety of clofilium were consistent with potent K_V_10.1 inhibition (Table 1).

In the MD simulation of the K_V_10.1–imipramine complex, there was rotation of F468, which formed parallel-displaced π–π interactions and occluded the aromatic ring of imipramine. The F468C mutation reduced the inhibition of K_V_10.1 by imipramine by a factor of 5, which correlated well with the MD simulation results [11]. Three Y464 residues also form π–π interactions, one in a T-shaped orientation and two in a parallel-displaced orientation on the other aromatic ring of imipramine.

Mutation studies of the aromatic rings in the central cavity showed that the F468C mutation reduced astemizole inhibition by a factor of about 20 [11]. The interactions analyzed in the MD simulation identified high incidence of hydrophobic and aromatic interactions between the aromatic rings of astemizole and Y464 and F468. Most aromatic interactions were formed with the benzoimidazole moiety of astemizole and Y464 (24% occurrence), but there were also aromatic interactions of the anisole and fluorophenyl moieties with Y464 of other subunits. Throughout the simulation, different types of interactions between astemizole and Y464 were seen for more than 90% of the simulation time (Table 3). Hydrophobic interactions with F468 (36% occurrence) were identified less frequently than with Y464, and we did not observe rotation of the F468 side chain into the central cavity, as described above for clofilium, imipramine, and quinidine. Astemizole and MK-499 both formed hydrogen bonds with the hydroxyl group of Y464, which raises the interesting question of whether the Y464F mutation reduces the K_V_10.1 affinity of the two ligands.

MK-499 also formed several interactions with Y464/F468. The most frequent and diverse interactions were with Y464. There was no rotation of F468 in the central cavity, but rather there was rotation of Y464 (Figure 8D, Table 3). Y464 positioned itself under the piperidine ring and formed a cation–π interaction (61% occurrence) and a hydrogen bond with the hydroxyl group of MK-499 (26% occurrence). Although astemizole binds similarly to MK-499, there was no rotation of Y464. There was also an additional Y464 in close proximity to the piperidine ring of MK-499 that formed hydrophobic interactions with it throughout the simulation, as well as hydrogen bonding (66% occurrence) with the chroman oxygen (Table 3).

**Figure 10:**
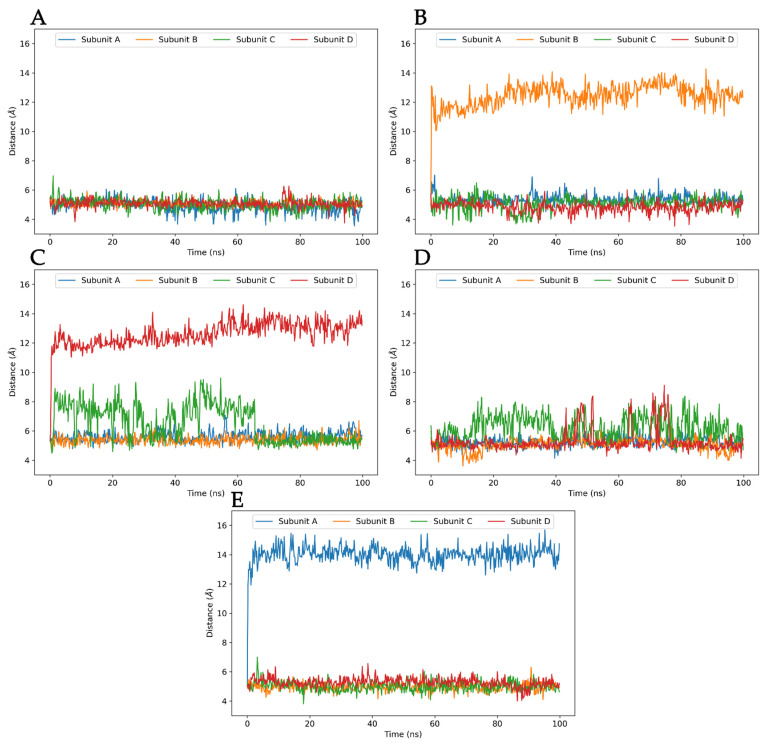
Distances between the aromatic rings of residues F359 and F468 of each subunit in the central cavity obtained by molecular dynamics simulations of astemizole (**A**), clofilium (**B**), imipramine (**C**), MK-499 (**D**), and quinidine (**E**) in complex with the homology model of the K_V_10.1. The distances increase for clofilium, imipramine, and quinidine, due to the rotation of F468 in the central cavity.

We analyzed the distances of the aromatic network formed with F359, Y464, F468, and the aromatic rings of clofilium, imipramine, and quinidine (Figure 10). The residue F359 that is located in segment S5 is analogous to F557 in the hERG channel, which has been reported to be important for the binding of several ligands [60]. Various binding modes have been proposed in which the ligands enter laterally from the central cavity to form an interaction with F557, with some of them moved almost completely out of the central cavity, possibly leaving enough space for the unobstructed passage of potassium ions through the selectivity filter [60,61]. We believe that perhaps instead of tight interactions between the ligands and F359 (F557 for hERG), the π–π network formed by the aromatic residues F359, Y464, F468, and the ligands is crucial. Of particular interest, some ligands can induce a conformational change of F468 into the central cavity, leading to disruption of the π–stacking of residues F359 and Y464. We observed this as an increase in the distance between the centers of the aromatic rings, from 4 Å to 8 Å (Figure 10). The rotation of Y464 for MK-499 showed significantly less interference with the π–π interactions of residues F359 and F468, compared to the rotation of F468. Such π–π networking was also observed in the hERG channel with residues F619, F557, Y652, and F656, and there was disruption of the π–π stacking between F557 and Y652 upon binding of cisapride [62]. The π–π network of K_V_10.1 lacks an additional aromatic ring that is present in hERG, as F619, which is instead M431 in K_V_10.1. One less aromatic residue in K_V_10.1 might be another reason the inhibitors showed a difference in inhibition of K_V_10.1 and hERG.

One of the most frequently detected interactions in all of these simulations (except for those with the aromatic rings in the central cavity) were hydrophobic interactions with A465 (Figure 8, Table 3). Interactions with at least one residue of A465 occurred in more than 90% of the simulation times for all of the ligands, except for MK-499, which showed a reduced frequency of 51%. There were also hydrogen bonds with S461 in the simulations of astemizole (51% occurrence) and MK-499 (32% occurrence). The formation of hydrogen bonds between the hydroxyl group on the 4-hydroxydihydropyran moiety of MK-499 and S461 (32% occurrence) or Y464 (25% occurrence) might lead to the potent binding affinity of MK-499. The study of the hERG channel showed that the MK-499 analog without the hydroxyl group on the dihydropyran moiety showed a reduced IC_50_ by a factor of 18, which highlights the potential involvement of the hydroxyl group in hydrogen bond formation. The A453S mutation reduced the inhibition of clofilium (6-fold increase in IC_50_), MK-499 (4-fold increase in IC_50_), and quinidine (2-fold increase in IC_50_), which demonstrated the importance of A453 for ligand binding, similar to what was seen in the MD simulations [41]. In the hERG channel, the equivalent residue is A653, which has been shown to be essential for normal channel function [63]. In the lower part of the central cavity, there were also interactions between T472 and all of the ligands. Astemizole and MK-499 formed hydrogen bonds with T472 for approximately 40% of the simulation time.

We also analyzed water-mediated hydrogen bonding in MD trajectories. In the case of clofilium, imipramine, and quinidine, hydrogen bond formation between the ligands and water molecules occurred in less than 1% of the simulation time; in the case of MK-499, interaction with a water molecule occurred in 27% of the simulation time; and in the case of astemizole, a water molecule formed hydrogen bonds in 42.6% of the simulation time. The latter was associated with the basic amine of the astemizole.

### 3.5. Creation of the Merged Structure-Based Pharmacophore Model

**Figure 11:**
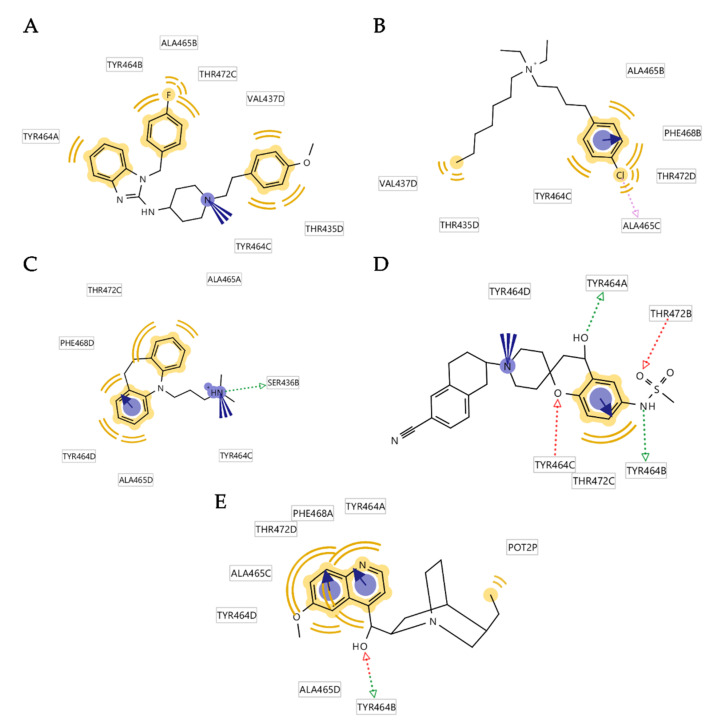
Two-dimensional projections of the frequently appearing pharmacophore models for astemizole (**A**), clofilium (**B**), imipramine (**C**), MK-499 (**D**), and quinidine (**E**) in complex with the homology model of the K_V_10.1; these were used in the creation of the final merged pharmacophore model. The pharmacophore features are: hydrophobic features, yellow; aromatic features, blue discs with arrows; hydrogen bond donors, green arrows; hydrogen bond acceptors, red arrows; positive ionizables, blue circles.

Our aim was to build a structure-based pharmacophore model that describes the binding modes of the simulated ligands to this homology model of the K_V_10.1 open pore conformation. The pharmacophore models generated in the MD trajectory for ligand interaction analysis (described above) were used to generate a merged pharmacophore model. Based on the occurrence of unique pharmacophore models among the 500 models from the MD trajectory, we selected the four most frequently appearing pharmacophore models per ligand, which were aligned and merged into structure-based pharmacophore models in LigandScout for each protein–ligand complex (Figure 11). The most frequently appearing pharmacophore models for the five ligands all show hydrophobic interactions in the lower part of the central cavity with residues Y464, A465, F468, and T472. All of the inhibitors except astemizole formed π–π interactions with Y464 or F468, while MK-499 and quinidine also formed hydrogen bonds with the hydroxyl group of Y464. The cation–π interaction was present in the pharmacophore models of astemizole, imipramine, and MK-499 (Figure 11).

The merged pharmacophore models generated for each of the K_V_10.1–ligand complexes were inspected and modified. Specifically, the overlapping pharmacophore features were interpolated, and all of the vector features were converted to nonvector sphere features. Five merged structure-based pharmacophore models for the five simulated ligands were then aligned. However, there was a problem with the quinidine pharmacophore model, as it could not be aligned with the merged models for the other four simulated ligands, and it was therefore excluded from the creation of the final merged pharmacophore model. The reason for the failed alignment of the quinidine model is due to the different binding mode of quinidine compared to the other simulated ligands. 

**Figure 12:**
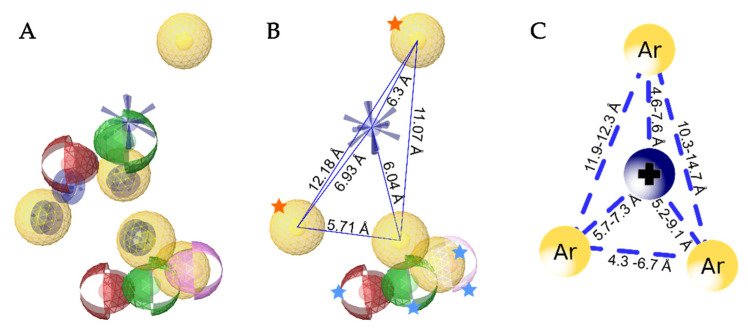
(**A**) Aligned and merged structure-based pharmacophore models from the MD simulations of astemizole, clofilium, imipramine, and MK-499 in complex with the homology model of the K_V_10.1. Pharmacophore features are: hydrophobic features, yellow spheres; aromatic features, blue discs; hydrogen bond donors, green spheres; hydrogen bond acceptors, red spheres; positive ionizable, blue star; halogen bond, pink sphere. (**B**) Simplified model used for validation, with hydrophobic and positive ionizable features connected by blue lines set as essential (only one feature marked with orange star is essential, the other one is optional), and the other features marked with blue stars set as optional. (**C**) Similarity of the present model is seen by the hERG pharmacophore model described by Cavalli et al. [15].

The key features for the alignment were a positively charged feature, two hydrophobic features and some of the exclusion spheres. The hydrophobic feature in the hydrophobic side pocket was shared by the pharmacophore models of astemizole, clofilium, and MK-499. The hydrophobic feature in the lower part of the central cavity in close proximity to the aromatic residue Tyr464 was shared by all the remaining four ligands. The final merged model of all of the four ligands had five hydrophobic features, one positively charged feature, three hydrogen bond donor features, two hydrogen bond acceptor features, four aromatic ring features, and one halogen bond feature (Figure 12A). None of the pharmacophore features was associated with the water molecule.

**Figure 13:**
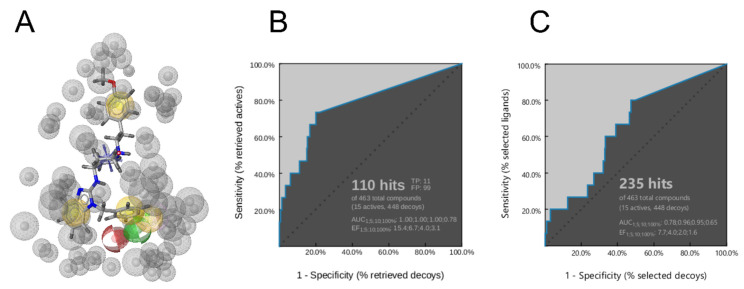
(**A**) Alignment of astemizole with the final three-dimensional merged pharmacophore model used for the virtual screening. Gray spheres show exclusion volumes. (**B**,**C**) Resulting ROC curves (blue) from the virtual screening of 463 compounds (15 K_V_10.1 inhibitors; 448 decoys [hERG inactive compounds based on ChEMBL for IC_50_ > 100 μM]) with the final merged pharmacophore model, with exclusion spheres (**B**) and without exclusion spheres (**C**). TP, true positives; FP, false positives; AUC, area under the curve; EF, enrichment factor.

To simplify the pharmacophore model, two hydrophobic features (Figure 12B, orange stars) were configured in a way that one is essential and the other is optional. The model was set up this way because some of the molecules do not bind in the hydrophobic side pockets (e.g., imipramine) or are more linear (e.g., MK-499). This increased the specificity of the model and maintained the retrieval of true active molecules. The third hydrophobic feature and the positive ionizable feature were selected as essential (Figure 12B, features without stars), while the remaining hydrophobic feature, and the hydrogen bond acceptor, hydrogen bond donor, and halogen bond features were set as optional (Figure 12B, blue stars). The final model (Figure 12B and Figure 13A) identified 11 of the 15 reported pore blockers of K_V_10.1 (Table S1). Quinidine, dronedarone, tetraethylammonium, and LY97241 did not fit the final pharmacophore model. This was expected for quinidine (described above), and for dronedarone and tetraethylammonium because of their sizes. Dronedarone was too large and clashed with the exclusion spheres, while tetraethylammonium was too small to fit the three essential pharmacophore features simultaneously. LY97241 is structurally very similar to clofilium, which was used to create the model, but was not retrieved by the model.

To test the model, we used a virtual library from two different sources. One library consisted of compounds with low or no hERG inhibition (Table S2), and therefore with low probability of inhibition of K_V_10.1. These compounds were retrieved from the ChEMBL database [31]. The second library of decoys was generated based on the 15 reported pore blockers of K_V_10.1 using the DUDE [52] decoy online server, which creates decoys with similar physicochemical properties but different molecular topologies. Our model performed well with a 15.4 enrichment factor at 1% of the library screened (Figure 13B). The model found 11 of 15 reported pore blockers active, and 99 of 448 hERG decoys active. Since the number of hERG decoys retrieved by the model is still quite high, the final merged pharmacophore model can be used as a filter in virtual screening and the hit list can then be rescreened with a set of more selective structure-based pharmacophore models derived from MD simulations of the individual hEAG1–ligand complexes. 

Our model resembles the hERG model proposed by Cavalli et al. [15], with the hydrophobic features at the same proposed distances from each other (Figure 12B,C). The main difference between the Cavalli et al. [15], model and ours is the additional exclusion spheres that restrict the space available for ligand binding and increase the selectivity of the model. When we tested our model in virtual screening without the exclusion spheres, it retrieved 223 of 448 hERG decoys (Figure 13C), and 508 of 850 decoys generated using the DUD-E server (Figure S5). Therefore, the selectivity of the model between the active and inactive compounds was poor. Dronedarone was the only additional active compound that was retrieved by the pharmacophore model without exclusion spheres.

Ligands that have been reported to inhibit K_V_10.1, but where the mechanism of action is not through the block of the potassium ion flux by binding to the central cavity of the channel, should therefore not fit our pharmacophore model. Our model was tested on such a virtual library (Supporting Information Table S3) that was constructed from ligands collected in a review article [17], with the addition of our newly identified set of K_V_10.1 inhibitors [53]. The pharmacophore model identified 16 of 61 active compounds. Chlorpromazine was among these, which is a modulator of the PAS domain. The structural similarity of chlorpromazine to imipramine might explain its inhibition of K_V_10.1 (which lacks the PAS domain) at higher voltages, which would explain why our model identified it as a hit [64]. The other molecules identified as hits were amiodarone and some purpurealidin analogs [65,66]. These were positioned in our model similar to imipramine, whereby the halogen substituents of the aromatic ring fit the two hydrophobic pharmacophore features, and the other aromatic ring protruded from the pharmacophore model in the direction that would represent entry into the central cavity. This mode of pharmacophore model matching was not observed for other active compounds in our virtual screening.

## 4. Conclusions

In this study, we used a combination of several molecular modeling techniques to analyze the binding mode of astemizole, clofilium, imipramine, MK-499, and quinidine in the pore of K_V_10.1, which is a promising target for anticancer drug development as it is expressed in more than 70% of tumors. With the aim being to understand the binding modes of ligands to the K_V_10.1 pore, we analyzed the potential of this binding site for the discovery of selective inhibitors.

The pharmacophore models created from the MD simulations were merged into a final MD-derived structure-based pharmacophore model that described the binding of K_V_10.1 inhibitors that bind to the central cavity. The pharmacophore model created distinguishes potential blockers from nonblockers, and it can be used to narrow down ligands that would bind in the central cavity of K_V_10.1. The model shows high similarity to the previously reported hERG pharmacophore model, which is consistent with the similar structures in the central cavity of the K_V_10.1 and hERG channels [15]. Our model includes exclusion spheres that greatly improved the selectivity and accuracy of the predictions, which thus define an important advantage of our model over previously published models for the hERG channel. The ligand–channel interactions in the MD trajectory that were identified were similar to those reported in the literature. Moreover, we observed an interesting rotation of F468 in a central cavity that disrupts the π–π network of aromatic residues that connect the pore domain to the voltage-sensor domain. Although the homology model of the open-pore conformation of K_V_10.1 was used in our study, the results agree well with the available experimental data. This demonstrates the advantages of using novel hERG structures to develop K_V_10.1 models for further development of novel K_V_10.1 inhibitors. However, new experimental K_V_10.1 structures in open pore conformation will inform us whether the assumed hydrophobic side pockets present in the hERG channel are also present in K_V_10.1.

The discovery of the disruption of the π–π network might represent an excellent starting point for further research to better understand ligand binding to K_V_10.1. Our pharmacophore model can be used to find new potential inhibitors of K_V_10.1, to help to increase the small number of currently known K_V_10.1 inhibitors. Furthermore, it can be used as a tool to distinguish ligands that do not bind in the central cavity, and so are more likely to inhibit K_V_10.1 in other ways, potentially increasing the likelihood that they will not inhibit the structurally similar hERG channel.

In addition, the molecular modeling method used in this work can be applied to other targets where the exact binding modes of the ligands are not known. As shown, it has several advantages over commonly used ligand-based methods, such as improved selectivity and accuracy of predictions, as well as insights into the disruption of important interaction networks in the protein structure.

## Figures and Tables

**Table 1:** Structures and inhibitory activities of the K_V_10.1 inhibitors investigated in this study.

Compound	Structure	K_V_10.1 IC_50_ [μM]	Cells and Technique Used in the Assay
Astemizole	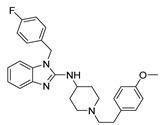	0.196	HEK-293 cells; whole-cell patch clamp [37]
		2.8 ± 0.1	*Xenopus* oocytes; two-electrode voltage clamp [11]
Clofilium	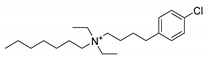	0.255 ± 0.035	CHO-K1 cells; whole-cell patch clamp [38]
		0.001 ± 0.001	*Xenopus* oocytes; inside-out patch clamp [38]
Imipramine	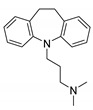	40.2 ± 3.0	*Xenopus* oocytes; two-electrode voltage clamp [11]
MK-499	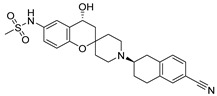	43.5 ± 4.7	*Xenopus* oocytes; whole-cell patch clamp [11]
Quinidine	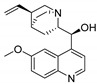	1.4 ± 0.1	CHO cells; whole-cell patch clamp [39]
		400 ± 200	*Xenopus* oocytes; two-electrode voltage clamp [40]
		2.1 ± 0.4	*Xenopus* oocytes; inside-out patch clamp [41]

**Table 2:** Scoring function scores of the K_V_10.1 inhibitors investigated in this study.

Compound	GlideScore [kcal/mol]
Astemizole	−11.459
Clofilium	−9.465
Imipramine	−8.995
MK-499	−9.631
Quinidine	−7.287

**Table 3:** Relative occurrences (%) of interactions with different residues in the K_V_10.1 binding site during the MD simulations for astemizole, clofilium, imipramine, MK-499, and quinidine (as indicated). Residues are labeled as A–D according to the subunits of K_V_10.1. The cells are colored according to the pharmacophore features: blue, aromatic; yellow, hydrophobic; red, hydrogen bond acceptor; green, hydrogen bond donor; cyan, positive ionizable; pink, halogen bond donor.

Astemizole																						
A465_A	A465_B		A465_C				S461_C		T435_D		T472_A		T472_C		Y464_A				Y464_B	Y464_C		V437_D
50	98		87				51		92		62		98	33	88	39		24	95	93	19	93
**Clofilium**																						
A465_A	A465_C		Y464_C				V437_D		F468_B		F468_C		T472_D									
94	97	28	93				90		84		93		99									
**Imipramine**																						
A465_B	A465_D		F468_D				S436_B		T472_C		Y464_A		Y464_B		Y464_D							
65	99		98		32		95		96		75	14	73		97		50					
**MK-499**																						
S461_A	T472_B		Y464_B				Y464_C		Y464_D		V437_D											
32	43		96	52		55	100	66	15	61	80											
**Quinidine**																						
A465_C	A465_D		F468_A				Y464_A		Y464_B		Y464_D											
82	92		99		79		93		87	51	94											

## Data Availability

The cryo-EM structures of K_V_10.1 (PDB ID code: 5K7L) and hERG (PDB ID code: 5VA1) are available in the Protein Data Bank at https://www.rcsb.org/ (accessed on 12 April 2019). Sequence alignment and script for creating homology model using Modeller, a typical file (prod.conf) for running MD simulations in NAMD, structures of K_V_10.1–ligand docking complexes (PDB format; shown in Figure 5), structures of K_V_10.1–ligand complexes from MD simulations (PDB format; shown in Figure 8) (ZIP).

## Supplementary Materials

The following are available online at https://www.mdpi.com/article/10.3390/ijms22168999/s1.
