# Supplementary material for: Molecular Dynamics-Derived Pharmacophore Model Explaining the Nonselective Aspect of KV10.1 Pore Blockers
Source: Int J Mol Sci. 2021 Aug 20;22(16):8999. doi: 10.3390/ijms22168999 (PMC8396485; doi:10.3390/ijms22168999)
Supplement: Supplementary file 1 [file ijms-22-08999-s001.zip › ijms-1347638-Supplementary materials.pdf]

Supplementary Material

Molecular dynamics-derived pharmacophore model explaining the nonselective aspect of Kv10.1 pore blockers

Žan Toplak [1], Franci Merzel [2], Luis A. Pardo [3], Lucija Peterlin Mašič [1] and Tihomir Tomašič [1,*]

[1] University of Ljubljana, Faculty of Pharmacy, 1000 Ljubljana, Slovenia; zan.toplak@ffa.uni-lj.si (Z.T.), lucija.peterlinmasic@ffa.uni-lj.si (L.P.M.), tihomir.tomasic@ffa.uni-lj.si (T.T.)
[2] Theory Department, National Institute of Chemistry, 1000 Ljubljana, Slovenia; franci.merzel@ki.si
[3] AG Oncophysiology, Max-Planck Institute for Experimental Medicine, 37075 Göttingen, Germany; pardo@em.mpg.de

* Correspondence: tihomir.tomasic@ffa.uni-lj.si; Tel.: +386-14769-556

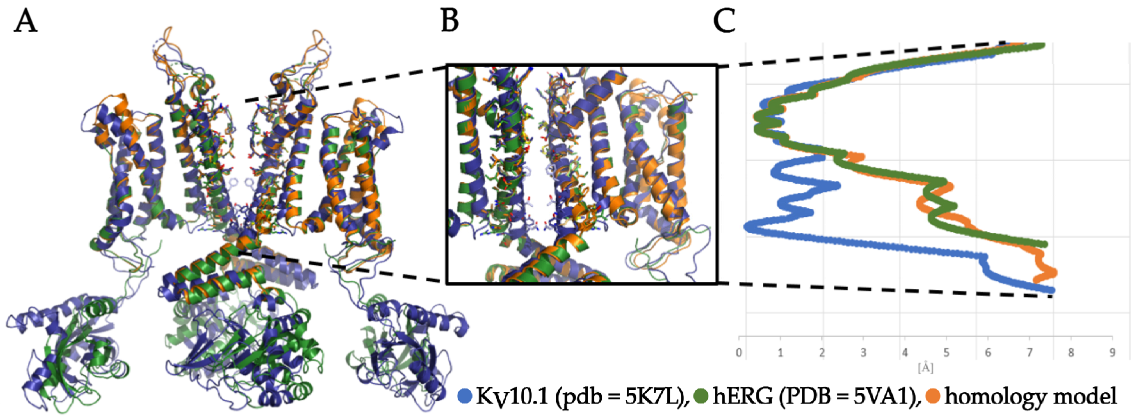

Figure S1. (**A**) Alignment of the closed conformation of Kv10.1 (PDB entry: 5K7L [1]; blue), the open conformation of hERG (PDB entry: 5VA1 [2]; green), and the best Kv10.1 homology model (orange) in the open conformation that was used in this study. Only two opposing subunits per channel are shown, for clarity. (**B**) The enlarged view of the pore region in (**A**) represented with sticks and illustrating the differences for the opened and closed channel conformations. (**C**) Measurements of the radius of the pore (Å) from the top of the selectivity filter using the HOLE program [3].

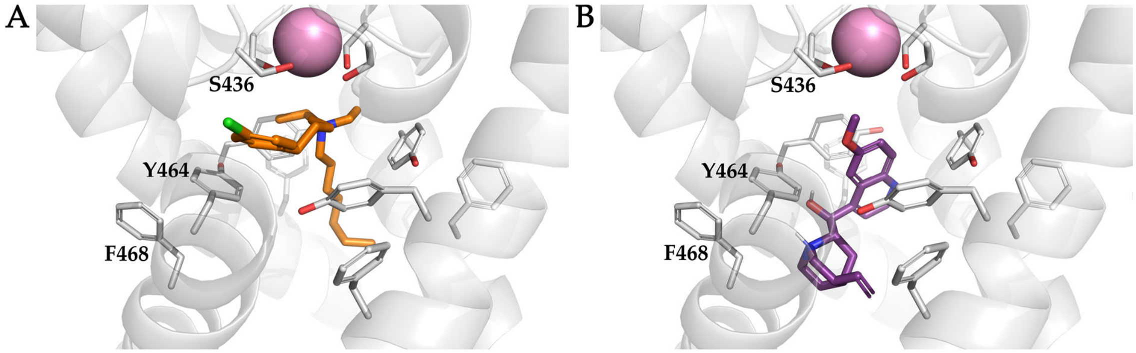

Figure S2. Alternative Glide [4] docking poses for clofilium (A) and quinidine (B) for those used in the molecular dynamics simulation studies.

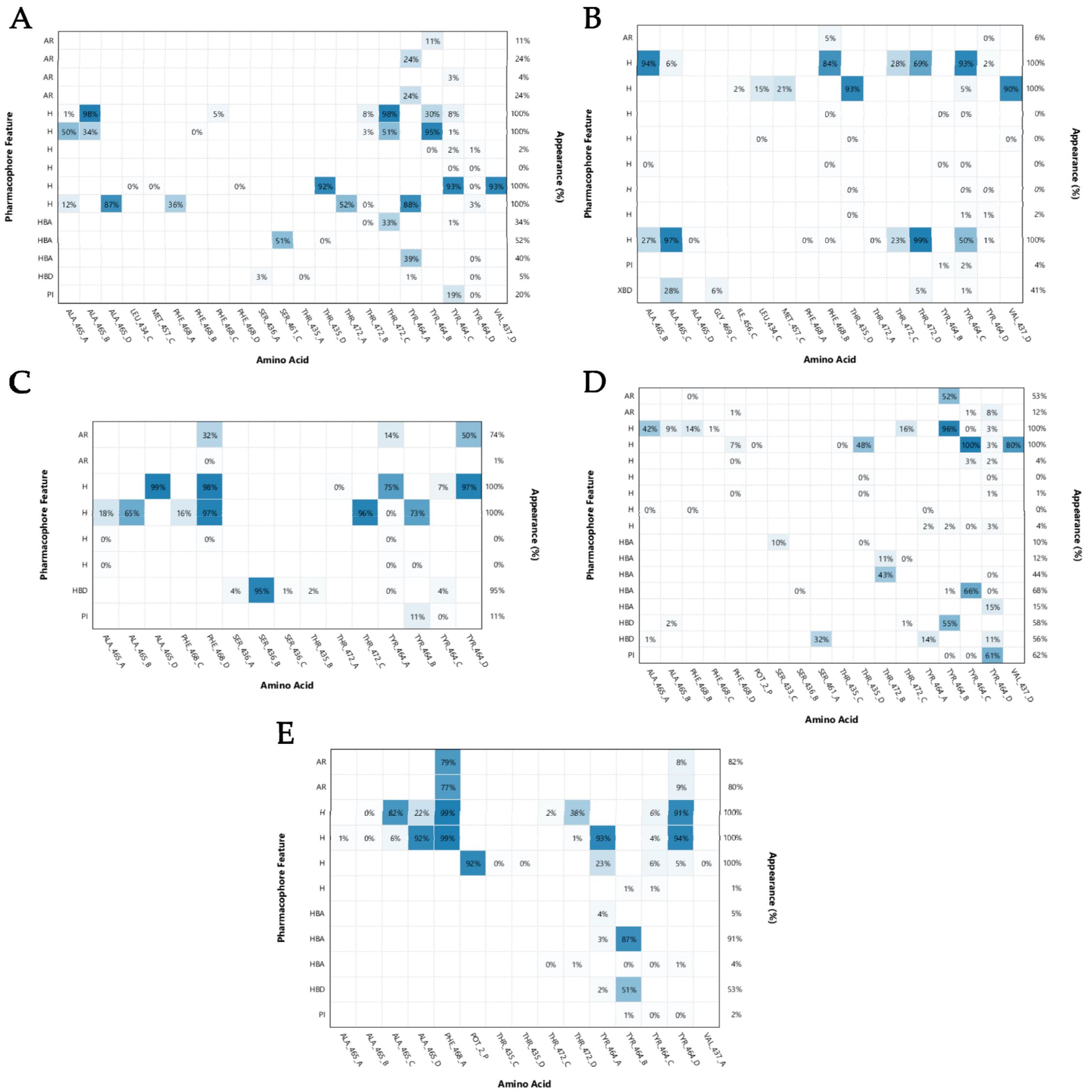

Figure S3. Interaction maps for astemizole (**A**), clofilium (**B**), imipramine (**C**), MK-499 (**D**), and quinidine (**E**) for various residues in the binding site of Kv10.1. The *y*-axis shows the individual interaction types based on the specific part of the ligand, for the overall occurrence of this interaction during the MD simulation: AR; aromatic interaction; H, hydrophobic interaction; HBD, hydrogen bond acceptor; HBA, hydrogen bond donor; PI, positive ionizable interaction; XBD, halogen bond donor. The *x*-axis shows the residue and the channel subunit that form the interaction, as the occurrence (%) in the grid, with the cells colored in different shades of blue based on the levels. The most frequent interactions were with Y464, A465, and F468, which were located in the lower part of the central cavity.

Astemizole

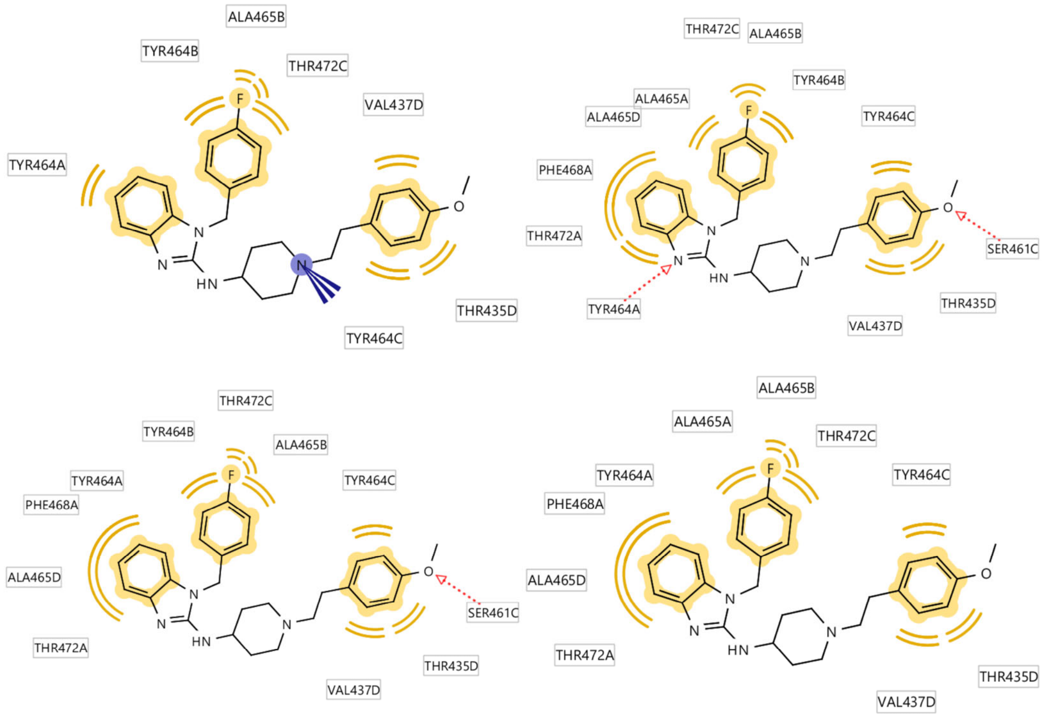

Clofilium

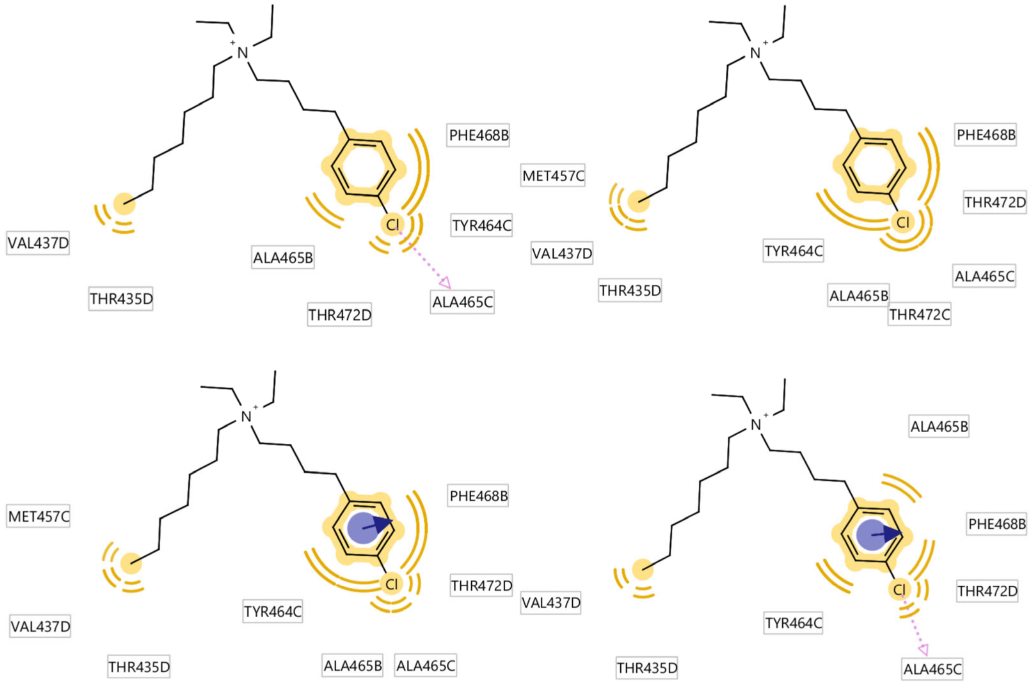

Imipramine

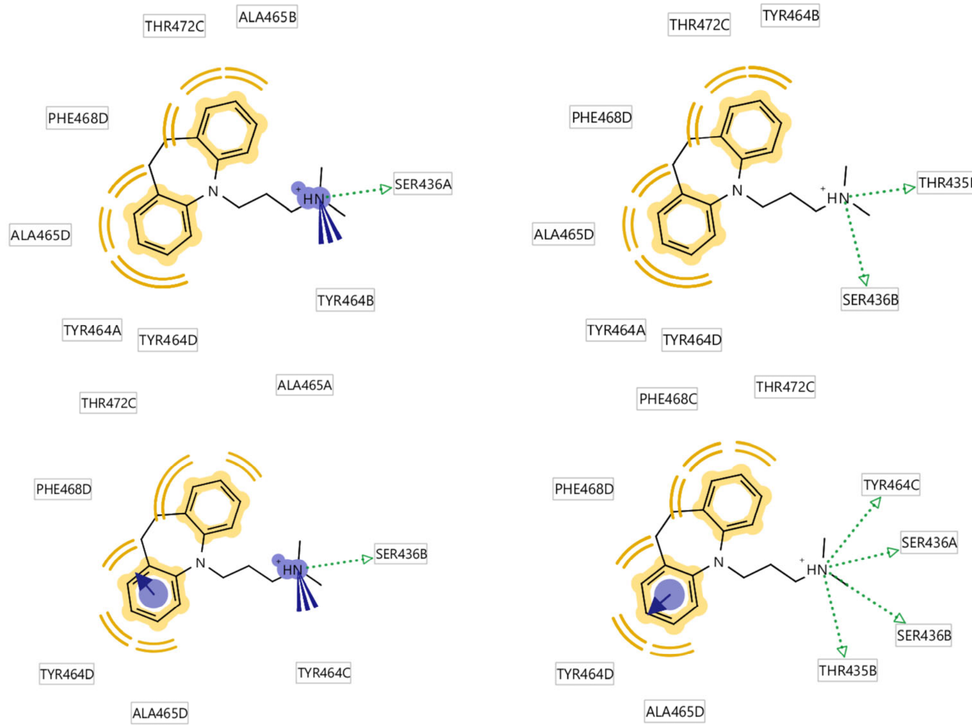

MK-499

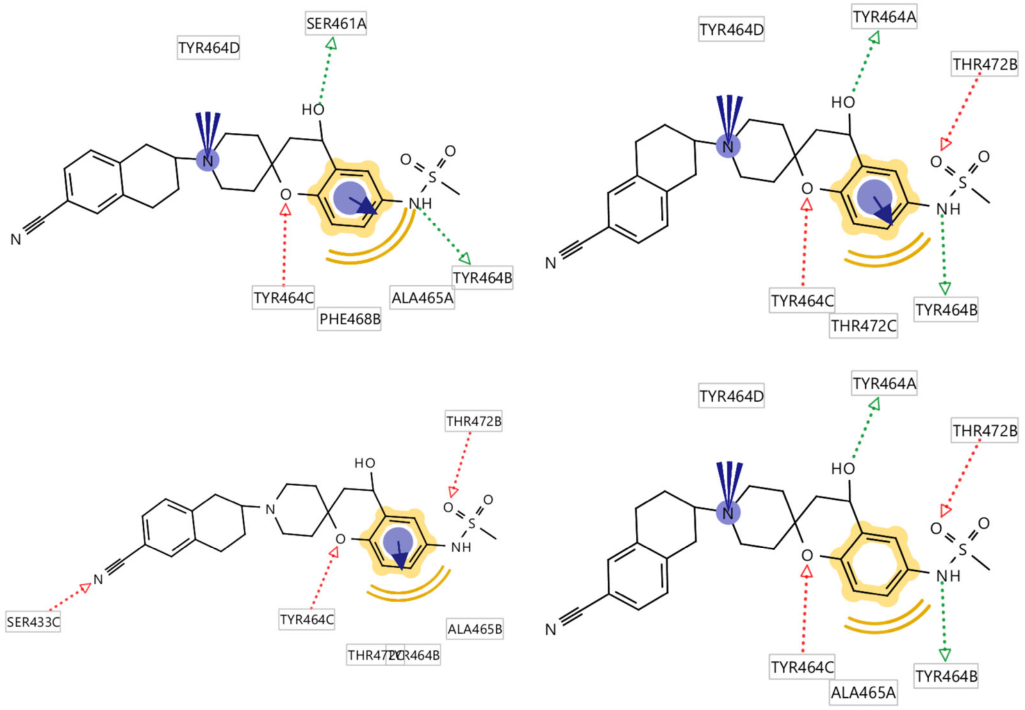

Quinidine

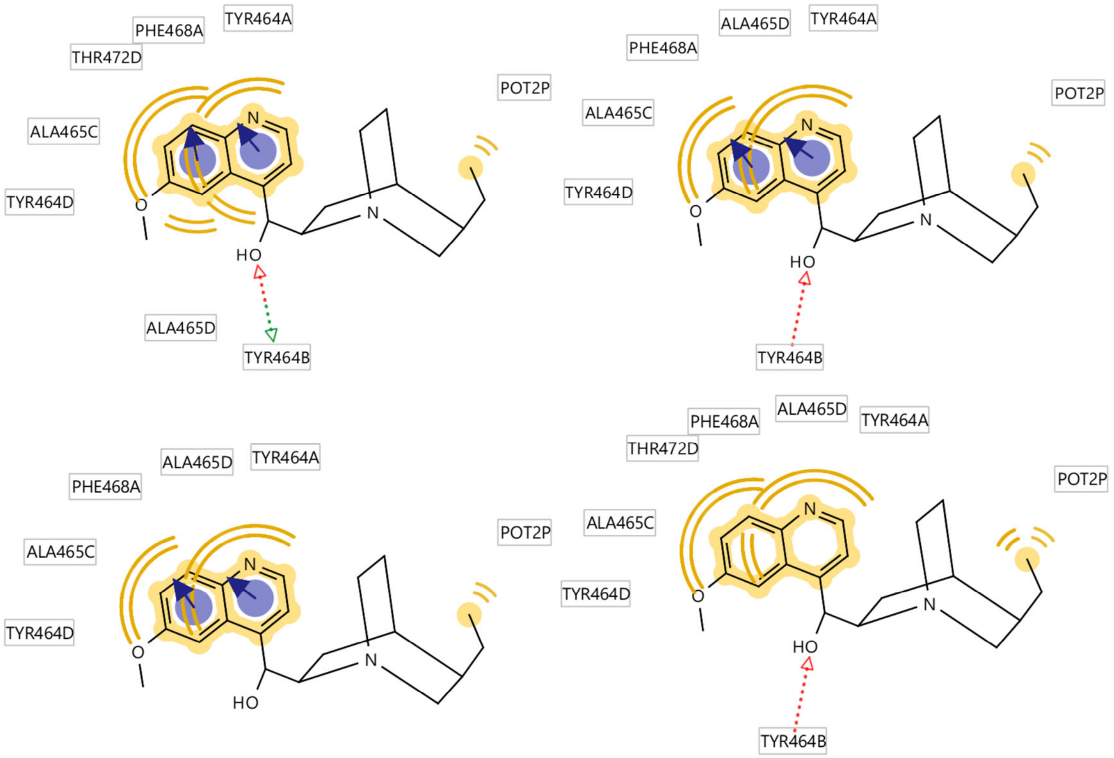

Figure S4. Two-dimensional projections of the four most frequently appearing pharmacophore models for astemizole, clofilium, imipramine, MK-499, and quinidine (as indicated) in complex with Kv10.1. Pharmacophore features: hydrophobic features, yellow; aromatic features, blue discs with arrows; hydrogen bond donors, green arrows; hydrogen bond acceptors, red arrows; halogen bonds, pink arrows; positive ionizables, blue circles.

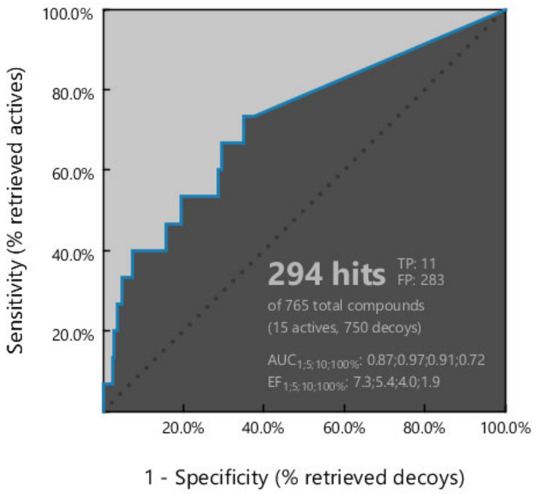

Figure S5. ROC curve (blue) from the virtual screening of 765 compounds (15 Kv10.1 pore blockers; 750 decoys generated using DUD-E online server). TP, true positives; FP, false positives; AUC, area under the curve; EF, enrichment factor.

Table S1. Structures of the Kv10.1 pore blockers used as the validation set for the final merged pharmacophore model [5].

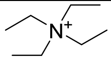 CC[N+](CC)(CC)CC	Tetraethylammonium (TEA)
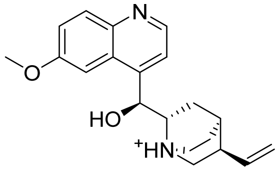 O[C@@H](C1=CC=NC2=CC=C(C=C12)OC)[C@@H]3C[C@H]4[C@@H](C=C)C[N@@H+]3CC4	Quinidine
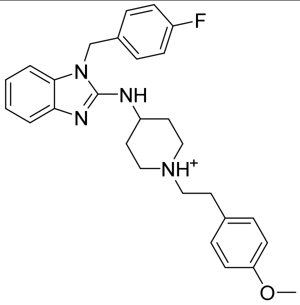 FC1=CC=C(CN2C(NC3CC[NH+](CC3)CCC4=CC=C(C=C4)OC)=NC5=CC=CC=C52)C=C1	Astemizole
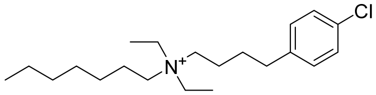 CCCCCCC[N+](CCCCC1=CC=C(Cl)C=C1)(CC)CC	Clofilium
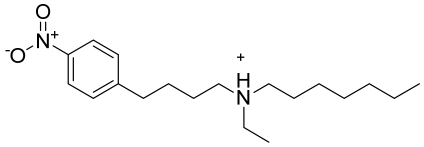 CCCCCCC[NH+](CC)CCCCC1=CC=C(C=C1)[N+]([O-])=O	LY97241
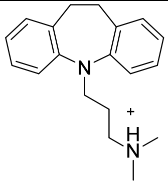 C[NH+](CCCN1C2=C(CCC3=C1C=CC=C3)C=CC=C2)C	Imipramine
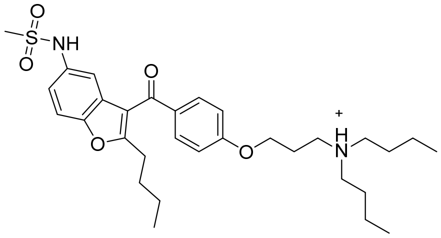 CCCC[NH+](CCCC)CCCOC1=CC=C(C=C1)C(C2=C(CCCC)OC3=C2C=C(NS(C)(=O)=O)C=C3)=O	Dronedarone

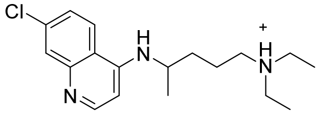<br>CC(NC1=CC=NC2=CC(Cl)=CC=C12)CCC[NH+](CC)CC	Chloroquine
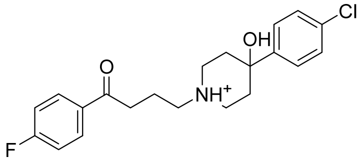<br>O=C(C1=CC=C(C=C1)F)CCC[NH+]2CCC(O)(CC2)C3=CC=C(C=C3)Cl	Haloperidol
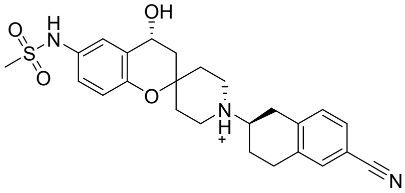<br>CS(=O)(NC1=CC2=C(C=C1)OC3(CC[N@@H+](CC3)[C@H]4CC5=C(CC4)C=C(C=C5)C#N)C[C@H]2O)=O	MK-499
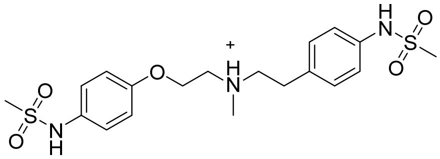<br>CS(NC1=CC=C(C=C1)OCC[NH+](CCC2=CC=C(C=C2)NS(C)(=O)=O)C)(=O)=O	Dofetilide
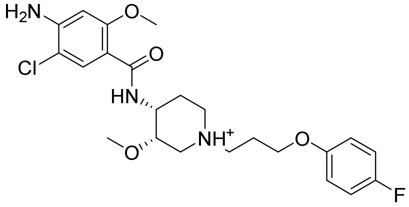<br>O=C(N[C@H]1[C@@H](OC)C[NH+](CCCOC2=CC=C(C=C2)F)CC1)C3=C(C=C(C(Cl)=C3)N)OC	Cisapride
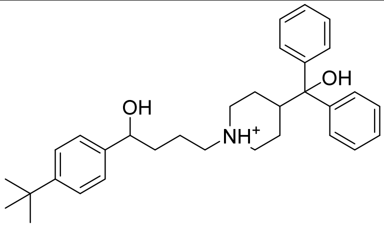<br>CC(C1=CC=C(C=C1)C(O)CCC[NH+]2CCC(CC2)C(O)(C3=CC=CC=C3)C4=CC=CC=C4)(C)C	Terfenadine
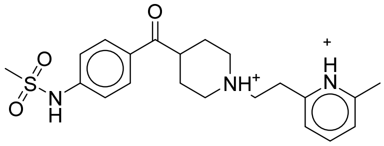<br>Cc1cccc(CC[NH+]2CCC(CC2)C(c3ccc(NS(=O)(C)=O)cc3)=O)n1	E4031
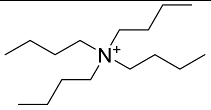<br>CCCC[N+](CCCC)(CCCC)CCCC	Tetrabutylammonium (TBA)

Table S2. Compounds with low (IC_{50} >100 μM) or no hERG inhibition, and therefore with low probability of inhibiting $K_V10.1$, as retrieved from the ChEMBL database (Target CHEMBL ID - CHEMBL240) [6] and filtered using the KNIME Analytics platform [7].

SMILES	Name
Nc1ccncc1	CHEMBL284348
CC(C(Cl)(Cl)Cl)(O)C	CHEMBL1439973
C[NH+]1CCC[C@H]1c1cccnc1	CHEMBL3
C[NH+]1CCCC1c1cccnc1	CHEMBL440464
COC(=O)C1=CCC2[NH+](C1CC2)C	CHEMBL412663
COC(=O)C1=CC[C@H]2[NH+]([C@@H]1CC2)C	CHEMBL451136
COC(=O)[C@H]1[C@@H](O)C[C@H]2[NH+]([C@@H]1CC2)C	CHEMBL1232472
CC[NH2+]CCc1cccc(c1)C(F)(F)F	CHEMBL1671896
CCCC(C1(CC)C(=O)NC(=O)NC1=O)C	CHEMBL448
Nc1nnc(c(n1)N)c1cccc(c1Cl)Cl	CHEMBL741
N#Cc1ccc(n1C)c1ccc(cc1F)O	CHEMBL4095883
OC[C@@H]1O[C@@H](C[C@H]1O)n1cc(C)c(=O)[nH]c1=O	CHEMBL374731
CC[NH+](CCNC(=O)c1ccc(cc1)N)CC	CHEMBL640
CC[NH+](CC(=O)Nc1c(C)cccc1C)CC	CHEMBL79
CCC1(C(=O)NC(=O)NC1=O)c1ccccc1	CHEMBL40
O=c1[nH]oc(c1)[C@H]1CC[NH2+][C@H](C1)CC(C)(C)C	CHEMBL3287851
C[C@@H]1OC[C@]2([C@@H](C1)CS[C+](=N2)N)c1nccs1	CHEMBL4214785
C[C@@H]1OC[C@]2([C@@H](C1)CS[C+](=N2)N)c1cccs1	CHEMBL4204215
NS(=O)(=O)c1ccc(cc1)OCC[NH+]1CCCC1	CHEMBL2024308
C[NH+]1CCCCC1C(=O)Nc1c(C)cccc1C	CHEMBL1087
c1ccc(cc1)Cc1ncc2c(n1)CC[NH2+]CC2	CHEMBL1770373
COC[C@H](C(=O)NCc1ccccc1)NC(=O)C	CHEMBL58323
NC(=O)N1c2ccccc2C=Cc2c1cccc2	CHEMBL108
F[C@@H]1CN([C@@H](C1)C#N)C(=O)[C@@H]([NH3+])Cc1cscn1	CHEMBL3127986
Cc1ccc2c(n1)ccc(c2)CC[NH+]1CCC[C@H]1C	CHEMBL1086033
OC1(CCCCC1)C(c1ccc(cc1)O)C[NH+](C)C	CHEMBL1118
N[C+]1=NCC2N1c1ccccc1Cc1c2cccc1	CHEMBL1106
O=C1N=[C+](NC1(c1ccccc1)c1ccccc1)O	CHEMBL16
CCc1cn(c2c1cc(Cl)cc2)c1ccc(cc1)F	CHEMBL300907
O=c1[nH]oc(c1)[C@H]1CC[NH2+][C@H](C1)Cc1ccccc1	CHEMBL3287849
N#Cc1cc(C)cc(c1)Oc1c(CC)[nH]nc1CC	CHEMBL576256
OC1CC2(C)C=CC(=O)C(=C2C2C1C(C)C(=O)O2)C	CHEMBL1673435
Fc1ccc(cc1)c1nn(cc1c1ccncc1)C	CHEMBL2420700
O=C(Cn1ccnc1[N+](=O)[O-])NCc1ccccc1	CHEMBL110
C[C@@H]1OC[C@]2([C@@H](C1)CS[C+](=N2)N)c1ncccc1F	CHEMBL4214603

CC[NH+](CCNC(=O)c1ccc(cc1)NC(=O)C)CC	CHEMBL1097
OC(c1ccccc1)(c1ccccc1)C1CC[NH2+]CC1	CHEMBL127508
Fc1ccc(cc1)n1cc(c2c1ccc(c2)Cl)C(O)C	CHEMBL52794
O=c1[nH]oc(c1)[C@H]1CC[NH2+][C@H](C1)CCc1ccccc1	CHEMBL3287854
NS(=O)(=O)c1ccc(cc1)OCCCC[NH+]1CCCC1	CHEMBL2024381
N#Cc1nc(NCC(C)C)c(c(n1)N1CCOCC1)N	CHEMBL450061
CCCc1cn(c2c1cc(Cl)cc2)c1ccc(cc1)F	CHEMBL52348
CC([NH+]1CCN(CC1)C(=O)N1CCc2c(C1)cccc2)C	CHEMBL219803
O[C@H]1C=C[C@@H]2[C@@]34[C@H]1Oc1c4c(C[C@H]2[NH+](CC3)C)ccc1O	CHEMBL70
CCCC[NH+]1CCCCC1C(=O)Nc1c(C)cccc1C	CHEMBL1200396
COc1cc(Cc2cnc(nc2N)N)cc(c1OC)OC	CHEMBL22
CCC(=O)c1cn(c2c1cc(Cl)cc2)c1ccc(cc1)F	CHEMBL53321
O=C(c1ccccc1)OC1CC2CCC(C1C(=O)O)[NH+]2C	CHEMBL1671895
C[C@H](c1nc2c(n1[C@H]1CCCOC1)c1cc[nH]c1nc2)O	CHEMBL2386653
CCCC[NH+]1CCCC[C@@H]1C(=O)Nc1c(C)cccc1C	CHEMBL2447962
CCC(c1cn(c2c1cc(Cl)cc2)c1ccc(cc1)F)O	CHEMBL53866
CCCC[NH+]1CCCC[C@H]1C(=O)Nc1c(C)cccc1C	CHEMBL1200749
Clc1ccc(cn1)Nc1n[nH]c2c1ccc(c2)C(F)(F)F	CHEMBL4093377
O=C1OCC(=C1c1ccccc1)c1ccc(cc1)[S+](=N)(C)[O-]	CHEMBL2029168
CCOc1nc(cc(c1C#N)N)C(=O)NCc1cccnc1	CHEMBL209534
CCC(c1cn(c2c1cc(Cl)cc2)c1ccc(cc1)F)CC	CHEMBL299390
F[C@H]1CCN(C1)C(=O)[C@H]([C@H]([C@@H]1CC[C@H](CC1)NC(=O)C)C)[NH3+]	CHEMBL237547
COc1ccc2c3c1O[C@@H]1[C@@]43CC[NH+]([C@H](C2)[C@@H]4C=C[C@@H]1O)C	CHEMBL485
C[NH+]1CCN(CC1)[C+]1=Nc2ccccc2Nc2c1cc(s2)C	CHEMBL715
FC(Oc1cc(ccc1F)c1cnn(c1)Cc1n[nH]cc1)F	CHEMBL3954438
N#C[C@@H]1CC[C@H](CC1)n1c(nc2c1cc[nH]c1nc2)[C@H](O)C	CHEMBL2386633
COc1cccc(c1)/C=C/C(=O)c1cc(OC)c(cc1O)OC	CHEMBL4071093
CN=[S+](c1ccc(cc1)C1=C(C(=O)OC1)c1ccccc1)(C)[O-]	CHEMBL1814813
O1CC[NH+](CC1)C[C@@H]1C[C@@H](C1)c1ccc(cc1)C[NH+]1CCCC1	CHEMBL2206288
OCc1ccc(cc1Cl)OC1CC[NH+](CC1)CC1CC[NH2+]CC1	CHEMBL272241
Cc1ccc2c(c1)cc([nH]2)C(=O)N1CCC(CC1)CC(O)(C)C	CHEMBL2413849
Fc1ccc(cc1F)Nc1n[nH]c2c1ncc(c2)OC(F)(F)F	CHEMBL4062193
N=[C+](c1cccs1)Nc1ccc2c(c1)CCN2C1CC[NH2+]CC1	CHEMBL2203713
N#CCC(=O)N1C[C@@H](C2(C1)CC2)N(c1ncnc2c1cc[nH]2)C	CHEMBL4279819
Clc1ccc2c(c1)N=[C+](N1CC[N+](CC1)([O-])C)c1c(N2)cccc1	CHEMBL1688
O=C(C1CCN(CC1)C(=O)N1CC[NH+](CC1)C(C)C)OC(C)(C)C	CHEMBL218410
N#CC[C@@H]1CC[C@H](CC1)n1c(nc2c1cc[nH]c1nc2)[C@H](O)C	CHEMBL2386635
OC(=O)c1ccc(cc1)C1=CC2(CC[NH2+]CC2)Oc2c1cccc2	CHEMBL494266
Fc1cc2c(cc1N1CC[NH2+]CC1)n(cc(c2=O)C(=O)O)C1CC1	CHEMBL8
Cc1nc(N)c2c(n1)n(nn2)C[C@@H]1OCC[NH+](C1)Cc1nccs1	CHEMBL2070735
O=S(=O)(c1ccccc1C(F)(F)F)Nc1cncc(c1)C(F)(F)F	CHEMBL3929544

O=C1COc2c(N1)nc(cc2)N1C[C@@H](C)OC[C@H]1c1ccccc1	CHEMBL2181928
O=S(=O)(c1ccccc1C(F)(F)F)Nc1ccc(nc1)C(F)(F)F	CHEMBL3924161
CC(NC(=O)NS(=O)(=O)c1ccc(cc1)OCC[NH+]1CCCC1)C	CHEMBL2024384
N#Cc1ccc(cc1)O[C@H]1[C@@H](C[C@@H]2[C@H]1CC2=O)[NH+]1CCC[C@H](C1)[NH3+]	CHEMBL4130214
COC(=O)C1=C(C)NC(=C(C1c1ccccc1[N+](=O)[O-])C(=O)OC)C	CHEMBL193
COc1ccc(cc1)n1cnc2c(c1=O)scc2c1ccccc1F	CHEMBL3330817
Fc1cc(F)c(cc1[C@H]1CCN(C[C@@H]1[NH3+])c1ccn2c(n1)nnc2)F	CHEMBL231294
OCC(NC(=O)c1nn(c2c1C[C@@H]1[C@H]2C1)c1ccc(cc1F)F)(C)C	CHEMBL3354952
COc1ccc2c(c1)c(CC(=O)O)c(n2C(=O)c1ccc(cc1)Cl)C	CHEMBL6
CCn1cc(C(=O)O)c(=O)c2c1c(F)c(c(c2)F)N1CC[NH2+]C(C1)C	CHEMBL561
CC([NH+]1CCN(CC1)C(=O)N1CCC(CC1)NC(=O)C1CCCC1)C	CHEMBL220407
COc1cc2CN(CCc2cc1OC)C(=O)N1CC[NH+](CC1)C(C)C	CHEMBL218992
N#Cc1cccc2c1CCN2C(=O)[C@](COc1ccc(cc1)F)(O)C	CHEMBL3238274
CNC(=O)c1ccc(cc1)C1=CC2(CC[NH2+]CC2)Oc2c1cccc2	CHEMBL551413
OC(=O)CNC(=O)c1c(O)cc(n2c1ncn2)CCc1ccccc1	CHEMBL4166742
CC(NC(=O)NS(=O)(=O)c1ccc(cc1)OCCC[NH+]1CCCC1)C	CHEMBL2024395
Fc1ccc(cc1)C(=O)N1CCn2c([C@H]1C)nnc2c1snc(n1)C	CHEMBL3608680
C[C@H](c1nc2c(n1[C@H]1CC[C@H](C1)[NH2+]CC(F)(F)F)c1cc[nH]c1nc2)O	CHEMBL2385096
C[NH+]1CCN(CC1)c1c(F)cc2c3c1OC[C@@H](n3cc(c2=O)C(=O)O)C	CHEMBL33
C[NH+]1CCN(CC1)c1c(F)cc2c3c1OCC(n3cc(c2=O)C(=O)O)C	CHEMBL4
F[C@H]1CCN(C1)C(=O)[C@H]([C@H](c1ccc(cc1)c1ccc(=O)n(c1)C)C)[NH3+]	CHEMBL206383
CCN(c1ccc(c2c1sc(n2)NC(=O)c1ccnn1C)OC)C(=O)C	CHEMBL1170367
OC[C@H](Cn1c(C#N)c(c2ccccc2)c2c(c1=O)ccc(c2)OC)O	CHEMBL216894
O=C(N1CCC(C1)(F)F)[C@H]1[NH2+]C[C@H](C1)[NH+]1CCN(CC1)c1ncccn1	CHEMBL515387
CC(NC(=O)NS(=O)(=O)c1ccc(cc1)OCCCC[NH+]1CCCC1)C	CHEMBL2021970
CCOc1nc(cc(c1C#N)N)C(=O)NCc1ccc(cc1)S(=O)(=O)C	CHEMBL424872
O=C1N(C[C@@H]([C@@H]1C(=O)N1CCCC1)[NH3+])C(=O)c1cnc2c(n1)cccc2	CHEMBL428936
OC(=O)C[C@@H]1CC[C@H](CC1)c1ccc(cc1)c1cnc2n(c1N)ncc2	CHEMBL2165819
CC(NC(=O)NS(=O)(=O)c1ccc(cc1)OCCC[NH+]1CCCCC1)C	CHEMBL2024400
COc1cccc2c1[C@H](c1ccccc1)N(CC2)C(=O)C[NH2+]C[C@H](O)C	CHEMBL3601260
Fc1c(cc2c(c1C)c(=O)c(cn2C1CC1)C(=O)O)N1CC[NH2+]C(C1)C	CHEMBL583
CC(=O)Nc1nccc(c1)c1c(nc(n1C)[S+](C)[O-])c1ccc(cc1)F	CHEMBL1773422
CCc1ccc(nc1)C(=O)COc1ccc(cc1)CC1SC(=O)NC1=O	CHEMBL146624
OC(=O)COCC[NH+]1CC[NH+](CC1)C(c1ccc(cc1)Cl)c1ccccc1	CHEMBL1000
COc1c(N2CC[NH2+]C(C2)C)c(F)cc2c1n(cc(c2=O)C(=O)O)C1CC1	CHEMBL31
OCc1ccc(cc1Cl)OC1CC[NH+](CC1)CC1CC[NH+](CC1)CC(=O)O	CHEMBL406028
N#CCC[NH+]1CCC(CC1)C[NH+]1CCC(CC1)Oc1ccc(c(c1)Cl)CO	CHEMBL271766
C[C@H](c1nc2c(n1[C@@H]1CC[C@H](CC1)[NH2+]CC(F)(F)F)c1cc[nH]c1nc2)O	CHEMBL2386629
CNC(=O)c1cccc(c1)NC(=O)N1CCC(CC1)Oc1ccccc1Cl	CHEMBL469169
O=C([C@H]([C@@H](c1onc(n1)c1ccc(cc1Cl)S(=O)(=O)C)C)[NH3+])N1CCCC1	CHEMBL216835
N#Cc1ccccc1Cn1c(nc2c(c1=O)[nH]cc2Br)N1CCC[C@H](C1)[NH3+]	CHEMBL2023067

O=c1ccn2c(n1)ccc(n2)N1CC[C@@H]([C@H](C1)[NH3+])c1cc(F)c(cc1F)F	CHEMBL231396
COc1ccc2c(c1)CCN([C@@H]2C(C)C)C(=O)C[NH2+]CC1(O)CCCCC1	CHEMBL3593759
F[C@H]1CCN(C1)C(=O)[C@H]([C@H](c1ccc(cc1)c1ccc(=O)n(c1F)C)C)[NH3+]	CHEMBL205872
F[C@H]1CCN(C1)C(=O)[C@H]([C@H](c1ccc(cc1)c1cccc(c1)C(=O)O)C)[NH3+]	CHEMBL195755
N#CC1(CC1)NC(=O)[C@@H]1CCCC[C@H]1C(=O)N1CCN(CC1)c1nccs1	CHEMBL2070938
N#Cc1ccccc1Cn1c(nc2c(c1=O)cc([nH]2)C)N1CCC[C@H](C1)[NH3+]	CHEMBL2022464
C[C@H]([C@H]1C(=O)N2[C@@H]1[C@@H](C)C(=C2C(=O)O)S[C@@H]1C[NH2+][C@@H](C1)CNS(=O)(=O)N)O	CHEMBL491571
O=C(NS(=O)(=O)c1ccc(cc1)OCC[NH+]1CCCC1)Nc1ccccc1	CHEMBL2024385
O=C(NS(=O)(=O)c1ccc(cc1)OCC[NH+]1CCCC1)NC1CCCCC1	CHEMBL2024386
CC(NC(=O)NS(=O)(=O)c1ccc(cc1)OCCCC[NH+]1CCCCC1)C	CHEMBL2021975
N#Cc1ccccc1Cn1c(nc2c(c1=O)cc([nH]2)Br)N1CCC[C@H](C1)[NH3+]	CHEMBL2022467
N#Cc1ccc(cc1)O[C@H]1[C@@H](C[C@H]2[C@]1(C)CCC(=O)C2)[NH+]1CCC[C@H](C1)[NH3+]	CHEMBL4127600
O=C(c1cnn(c1Cl)C)N1CCC(CC1)Nc1cc(=O)[nH]c2c1cccc2	CHEMBL3290766
N#Cc1cnc(cn1)N1CCC(CC1)Nc1c(cnc2c1cc[nH]2)C(=O)N	CHEMBL3593775
COc1ccc2c(c1)CCN([C@H]2C(C)C)C(=O)C[NH2+]CC1(O)CCCCC1	CHEMBL3593760
CCCCCCCCCCCCCCCCOP(=O)(OCC[N+](C)(C)C)[O-]	CHEMBL125
N#Cc1ccc(nn1)N1CCC(CC1)Nc1c(cnc2c1cc[nH]2)C(=O)N	CHEMBL3593776
N#Cc1ccc(cn1)S(=O)(=O)N[C@@H](c1nc2c(n1CC)cc(nc2)C1CC1)C	CHEMBL3605558
Fc1cccc(c1F)C[NH+]1CCN(CC1)c1ccc2n(n1)c(nn2)C(F)(F)F	CHEMBL2346969
N#Cc1ccc(nc1)N1CC[C@@H]([C@@H](C1)F)Nc1c(cnc2c1cc[nH]2)C(=O)N	CHEMBL3593778
O=C(NS(=O)(=O)c1ccc(cc1)OCCC[NH+]1CCCC1)NC1CCCCC1	CHEMBL2024397
CCC(=C(c1ccc(cc1)O)c1ccc(cc1)/C=C/C(=O)O)c1ccccc1	CHEMBL195515
COC=C([C@H]1C[C@@H]2[NH+](C[C@@H]1CC)CC[C@@]12C(=O)Nc2c1cccc2)C(=O)OC	CHEMBL519266
COC([C@@H](C(=O)O)Oc1nc(C)cc(n1)C)(c1ccccc1)c1ccccc1	CHEMBL1111
CCN(C(=O)c1ncc(cn1)C1=CC2(CC[NH2+]CC2)Oc2c1cccc2)CC	CHEMBL562280
N#C[C@@H]1CCCN1C(=O)[C@@H]1C[NH+](C[C@@H]1[NH3+])Cc1ccc(cc1)c1nnc(o1)C	CHEMBL399726
COc1cc2nc(nc(c2cc1OC)N)N(CCCNC(=O)C1CCCO1)C	CHEMBL709
O=C(N1CCn2c(C1)nnc2C(F)(F)F)C[C@@H](Cc1cc(F)c(cc1F)F)[NH3+]	CHEMBL1422
OCc1cc(=O)n(cc1c1ccc(cc1)[C@@H]([C@@H](C(=O)N1CC[C@@H](C1)F)[NH3+])C)C	CHEMBL203169
FC(c1nc(NCc2ccc3c(c2)OCO3)nc(c1)Nc1ccncc1)(F)F	CHEMBL607259
N#C[C@@H]1CCCN1C(=O)C[NH2+]C(CNC(=O)c1cnc2n(c1)nc(c2)C)(C)C	CHEMBL1929387
Fc1ccc(cc1)[C@H]1Nc2cc(F)cc3c2c([C@@H]1c1ncnn1C)n[nH]c3=O	CHEMBL3137320
N#Cc1ccc(nc1)N1CC[C@H]([C@H](C1)F)Nc1c(cnc2c1cc[nH]2)C(=O)N	CHEMBL3593777
CC([NH+]1CCN(CC1)C(=O)N1CCC(CC1)C(=O)N1CC2CCC(C1)CC2)C	CHEMBL218834
O1CC[NH+](CC1)[C@@H]1CC[C@H](CC1)Nc1ncnc2c1c(c[nH]2)C1CCOCC1	CHEMBL4064608
Nc1ccc(cn1)c1ncc2c(c1)sc(n2)N1CCC(CC1)[NH+]1CCCCC1	CHEMBL575685
Oc1ccc(cn1)c1cnc2c(c1)sc(n2)N1CCC(CC1)[NH+]1CCCCC1	CHEMBL574393
N#Cc1ccc(cc1)Nc1nc(N)c(c(n1)Oc1c(C)cc(cc1C)C#N)Br	CHEMBL308954
C[C@@H]1[NH2+][C@H](C)CN(C1)c1c(F)c(N)c2c(c1F)n(cc(c2=O)C(=O)O)C1CC1	CHEMBL850
O=C(NS(=O)(=O)c1ccc(cc1)OCC[NH+]1CCCCC1)NC1CCCCC1	CHEMBL2024391
Nc1cnc(cn1)c1ncc2c(c1)sc(n2)N1CCC(CC1)[NH+]1CCCCC1	CHEMBL575453

Fc1ccc2c(c1)c(NC1CCN(CC1)C(=O)c1cnn(c1Cl)C)cc(=O)[nH]2	CHEMBL3290767
CS(=O)(=O)C1(CC1)c1cc(nc(n1)c1cccc2c1cc[nH]2)N1CCOCC1	CHEMBL2325704
Oc1ccc(cn1)c1ncc2c(c1)sc(n2)N1CCC(CC1)[NH+]1CCCCC1	CHEMBL578834
OC(=O)c1ccc(cc1)n1nc(nc1c1ccccc1O)c1ccccc1O	CHEMBL550348
O=C(c1n[nH]c(n1)Cc1ccccc1)N[C@H]1COc2c(N(C1=O)C)cccc2	CHEMBL4071864
[NH3+][C@H]1C[C@H](CC[C@@H]1c1cc(F)c(cc1F)F)[NH+]1Cc2c(C1)c1n(n2)cccn1	CHEMBL3806003
N#Cc1ccc(cc1)O[C@H]1[C@@H](C[C@H]2[C@]1(C)CCC(C2)(C)O)[NH+]1CCC[C@H](C1)[NH3+]	CHEMBL4126938
O=C(c1cnc(nc1C1CC1)N1CCOCC1)N[C@@H]1C2CC3CC1C[C@](C2)(C3)O	CHEMBL2158485
O=C(c1cnn(c1Cl)C)N1CCC(CC1)Nc1cc(=O)[nH]c2c1cc(F)c(c2)F	CHEMBL3290768
O=C(NS(=O)(=O)c1ccc(cc1)OCCCC[NH+]1CCCC1)NC1CCCCC1	CHEMBL2021972
CON=C1CN(CC21C[NH2+]C2)c1nc2N(CC(C(=O)c2cc1F)C(=O)O)C1CC1	CHEMBL1671899
O=C1N(C)CC(=O)N2C1Cc1c(C2c2ccc3c(c2)OCO3)[nH]c2c1cccc2	CHEMBL1671905
COc1c(c(F)cc2c1n(cc(c2=O)C(=O)O)C1CC1)N1C[C@@H]2[C@H](C1)CCC[NH2+]2	CHEMBL32
O=CN(C1(CCOCC1)CS(=O)(=O)N1CCC(CC1)CCc1c(C)noc1C)O	CHEMBL1784340
N#CC1(CC1)NC(=O)[C@@H]1CCCC[C@H]1C(=O)N1CCN(CC1)c1sc(c(n1)C)C	CHEMBL2070939
O=C1N(C)CC(=O)N2[C@@H]1Cc1c([C@H]2c2ccc3c(c2)OCO3)[nH]c2c1cccc2	CHEMBL779
CCC1(C)NC(=O)c2c(NC1=O)c(Cl)cc(c2)S(=O)(=O)Nc1ccc(cc1F)F	CHEMBL3134098
OCC(c1ccc(cc1)Cl)NC(=O)C1([NH3+])CCN(CC1)c1ncnc2c1cc[nH]2	CHEMBL2325737
O=C(NS(=O)(=O)c1ccc(cc1)OCCC[NH+]1CCCCC1)Nc1ccccc1	CHEMBL2024401
O=C(NS(=O)(=O)c1ccc(cc1)OCCCC[NH+]1CCCC1)Nc1ccccc1	CHEMBL2021971
O=C(NS(=O)(=O)c1ccc(cc1)OCCC[NH+]1CCCC1)Nc1ccc(cc1)C	CHEMBL2021983
O=C(NS(=O)(=O)c1ccc(cc1)OCC[NH+]1CCCC1)Nc1cc(Cl)ccc1Cl	CHEMBL2024387
CCCSc1nc(ccc1C(=O)NC1CCCCC1)N1CCC[C@H](C1)CC(=O)O	CHEMBL2153191
N#Cc1cc(ccc1Oc1ccc(c(c1)Cl)CO)S(=O)(=O)Nc1ccc(cn1)F	CHEMBL3688197
N#CC(=C1CCN(CC1)c1ccc(cc1F)N1C[C@@H](OC1=O)Cn1ccnn1)C	CHEMBL2324519
N#Cc1ccc(cn1)S(=O)(=O)N[C@@H](c1nc2c(n1CC)cc(nc2)C(F)(F)F)C	CHEMBL3605557
N#CC1(CC1)NC(=O)[C@@H]1CCCC[C@H]1C(=O)N1CCN(CC1)c1scc(n1)CC	CHEMBL2070940
C[C@@H]1COCCN1c1cc(nc(n1)c1ccnc2c1cc[nH]2)C1(CC1)[S@@](=O)(=N)C	CHEMBL4285417
Clc1cc(Cl)cc(c1)c1ncccc1[C@@H](C(c1cccnc1)c1cccnc1)O	CHEMBL4104525
O=C(c1ccc2c(c1)[nH]c([nH+]2)C)N1CCC2(CC1)CC(=O)c1c(C2)cnn1C(C)C	CHEMBL3359265
C[NH+]1CCC(CC1)S(=O)(=O)c1c(Cl)ccc(c1O)NC(=O)Nc1cccc(c1Cl)F	CHEMBL3819512
O=C(c1ccccc1)CN1C(=C(O)c2c(S1(=O)=O)cccc2)C(=O)c1ccccc1	CHEMBL600686
C[C@@H]1C[NH+](CCN1S(=O)(=O)c1ccc(cc1)[C@](C(F)(F)F)(O)C)CC1(CC1)C(=O)N	CHEMBL489500
NC(=O)C(c1ccc(cc1)Cl)NC(=O)C1([NH3+])CCN(CC1)c1ncnc2c1cc[nH]2	CHEMBL2325730
C[C@@H]1C[NH+](CCN1S(=O)(=O)c1ccc(cc1)C(C(F)(F)F)(O)C)CC1(CC1)C(=O)N	CHEMBL465955
O=C(NS(=O)(=O)c1ccc(cc1)OCCCC[NH+]1CCCCC1)Nc1ccccc1	CHEMBL2021976
COC(=O)CCC[NH+]1CCC(CC1)C[NH+]1CCC(CC1)Oc1ccc(c(c1)Cl)CO	CHEMBL402450
F[C@H]1CCN(C1)C(=O)[C@H]([C@@H](c1onc(n1)c1ccc(cc1)S(=O)(=O)C)CC1CC1)[NH3+]	CHEMBL375442
O=C(NS(=O)(=O)c1ccc(cc1)OCCC[NH+]1CCCC1)Nc1cc(Cl)ccc1Cl	CHEMBL2024398
O=C(NS(=O)(=O)c1ccc(cc1)OCCC[NH+]1CCCCC1)Nc1ccc(cc1)C	CHEMBL2021991
C[C@@H]1C[NH+](CCN1S(=O)(=O)c1ccc(cc1)[C@@](C(F)(F)F)(O)C)CC1(CC1)C(=O)N	CHEMBL460962

O=C(NS(=O)(=O)c1ccc(cc1)OCC[NH+]1CCCCC1)Nc1cc(Cl)ccc1Cl	CHEMBL2024392
N#CC1(CC1)NC(=O)[C@@H]1CCCC[C@H]1C(=O)N1CCN(CC1)c1scc(n1)C1CC1	CHEMBL2070941
C[C@@H]1CCCC[NH+]1CCC[NH2+]Cc1ccc(cc1)C[NH2+]CCC[NH+]1CCCC[C@@H]1C	CHEMBL2170299
C[C@@H]1CCCC[NH+]1CCC[NH2+]Cc1ccc(cc1)C[NH2+]CCC[NH+]1CCCC[C@H]1C	CHEMBL2170444
O=C(NS(=O)(=O)c1ccc(cc1)OCCCC[NH+]1CCCCC1)NC1CCCCC1	CHEMBL2021977
OCC[C@@H](c1ccc(cc1)Cl)NC(=O)C1([NH3+])CCN(CC1)c1ncnc2c1cc[nH]2	CHEMBL2325741
OC(=O)/C=C/c1ccc(cc1)Cc1c(c2ccccc2)c(=O)oc2c1ccc(c2)O	CHEMBL3427385
OCCC(c1ccc(cc1)Cl)NC(=O)C1([NH3+])CCN(CC1)c1ncnc2c1cc[nH]2	CHEMBL2325738
CC[NH+](CC(=O)N1CCC[C@H]1c1cc(c(s1)C)C(=O)Nc1ncc(s1)F)CC(=O)O	CHEMBL4283796
O=C(C1CC1)Nc1nc2n(n1)c(ccc2)c1ccc(cc1)C[NH+]1CCS(=O)(=O)CC1	CHEMBL3301607
C=CC(=O)N[C@@H]1CN(C[C@H]1F)c1nc(Nc2cn(nc2OC)C)c2c(n1)n(C)cn2	CHEMBL3989970
O=C(N1CCn2c(C1)nnc2C(F)(F)F)C1=CC[C@@H]([C@H](C1)[NH3+])c1cc(F)c(c(c1)F)F	CHEMBL437341
Clc1ccc(c(c1)NC(=O)NS(=O)(=O)c1ccc(cc1)OCCCC[NH+]1CCCC1)Cl	CHEMBL2021973
COc1ccc(cc1)NC(=O)NS(=O)(=O)c1ccc(cc1)OCCC[NH+]1CCCCC1	CHEMBL2021989
N#CC1(CC1)NC(=O)[C@@H]1CCCC[C@H]1C(=O)N1CCN(CC1)c1nc2c(s1)cccn2	CHEMBL2071095
CCOC[C@@H](C(=O)Nc1ccccn1)Oc1ncnc2c1cnn2c1ncccc1Cl	CHEMBL3221501
O=C(c1ccc(c(c1)NC(=O)c1cnn(c1N)c1ccccc1F)C)Nc1ccon1	CHEMBL1290746
CC1CCC(CC1)CCCC[NH+]1CCC(CC1)C(c1ccccc1)(c1ccccc1)O	CHEMBL571174
COCCOCC#Cc1scc(c1)c1n[nH]c2c1Cc1c2ccc(c1)Cn1cncn1	CHEMBL445404
CCC1OC(=O)[C@H](C1)[NH+]1CCC(CC1)C[NH+]1CCC(CC1)Oc1ccc(c(c1)Cl)CO	CHEMBL271971
N#CC1(CC1)NC(=O)[C@@H]1CCCC[C@H]1C(=O)N1CCN(CC1)c1nc2c(s1)cncc2	CHEMBL2070950
CNC(=O)CCCN(C(=O)c1ccc2c(c1)c1C[C@@H](CCc1n2C)C1CCOCC1)C	CHEMBL2029729
COc1cc(ccc1OC)C(NC(=O)C1([NH3+])CCN(CC1)c1ncnc2c1cc[nH]2)C	CHEMBL2325991
COC[C@@H](C(=O)Nc1ccc(cn1)C)Oc1ncnc2c1cnn2c1ncccc1Cl	CHEMBL3221483
N#CC1(CC1)NC(=O)[C@@H]1CCCC[C@H]1C(=O)N1CCN(CC1)c1nc2c(s1)ccnc2	CHEMBL2070951
O=C(c1cnn(c1C(C)(C)C)c1ccc(cc1)C(=O)O)NC1C2CC3CC1CC(C2)C3	CHEMBL2177609
C[NH+]1CCN(CC1)c1ccc2c(c1)c(=O)n(cn2)c1cc(ccc1C)C(=O)NC1CC1	CHEMBL2031465
CN(c1ccc(cc1)NC(=O)NS(=O)(=O)c1ccc(cc1)OCCC[NH+]1CCCC1)C	CHEMBL2021985
O=C(NS(=O)(=O)c1ccc(cc1)OCC[NH+]1CCCC1)Nc1ccc(cc1)C(F)(F)F	CHEMBL2024388
O=C(NS(=O)(=O)c1ccc(cc1)OCCC[NH+]1CCCC1)Nc1ccc(cc1)C(=O)C	CHEMBL2021982
Clc1ccc(c(c1)NC(=O)NS(=O)(=O)c1ccc(cc1)OCCC[NH+]1CCCCC1)Cl	CHEMBL2021967
CC[NH+]1CCC(CC1)S(=O)(=O)c1c(Cl)ccc(c1O)NC(=O)Nc1cccc(c1Cl)F	CHEMBL3818331
N#CC1(CC1)NC(=O)[C@@H]1CCCC[C@H]1C(=O)N1CCN(CC1)c1nc2c(s1)COCC2	CHEMBL2071097
Fc1ccc(cc1)C1CNC(=O)C21CC[NH+](CC2)C1CCCCC1(O)c1ccccc1	CHEMBL378253
OC(=O)/C=C/c1ccc(cc1)Oc1c(c(=O)oc2c1cccc2)c1ccc(cc1C)F	CHEMBL3427414
OC(=O)CNC(=O)[C@H](C[NH+]1CC[C@@]([C@H](C1)C)(C)c1cccc(c1)O)Cc1ccccc1	CHEMBL270190
CO[C@@H](c1cc(Cl)c2c(c1Cl)C(=O)N(CC2)Cc1c(OC)cc([nH]c1=O)C)C1COC1	CHEMBL4080228
N#CC1(CC1)NC(=O)[C@@H]1CCCC[C@H]1C(=O)N1CCN(CC1)c1nc2c(s1)nccc2	CHEMBL2070949
CC#CCn1c(cc(=O)n(c1=O)Cc1ccc2c(n1)cc(cc2)Cl)N1CCC[C@H](C1)[NH3+]	CHEMBL2425893
N#Cc1cnn2c1nc(Nc1ccc(c(c1)NC(=O)C)N(CC[NH3+])C)cc2NC1CC1	CHEMBL3103192
O=C(NS(=O)(=O)c1ccc(cc1)OCCCC[NH+]1CCCCC1)Nc1cc(Cl)ccc1Cl	CHEMBL2021978

O=C(NS(=O)(=O)c1ccc(cc1)OCCC[NH+]1CCCC1)Nc1cccc(c1)C(F)(F)F	CHEMBL2021981
O=C(NS(=O)(=O)c1ccc(cc1)OCCC[NH+]1CCCC1)Nc1cccc2c1cccc2	CHEMBL2021984
Fc1ccc(cc1)CNC(=O)c1nc(n(c(=O)c1O)C)C(NC(=O)c1nnc(o1)C)(C)C	CHEMBL254316
O=C(NS(=O)(=O)c1ccc(cc1)OCCC[NH+]1CCCCC1)Nc1ccc(cc1)C(=O)C	CHEMBL2021990
O=C(Cn1c(Cc2c(Cl)cccc2Cl)nc2c1cccc2)Nc1cc(ccc1C)C(C)C	CHEMBL3948914
OCc1ccc(cc1Cl)OC1CC[NH+](CC1)CC1CCN(CC1)c1ccccc1C(=O)O	CHEMBL409449
O=C([C@H]([C@@H](C(=O)N1CCC(C1)(F)F)[NH3+])c1ccc(cc1)c1ccc2n(c1)ncn2)N(C)C	CHEMBL209064
O=C([C@H]([C@@H](C(=O)N1CCC(C1)(F)F)[NH3+])c1ccc(cc1)c1ccn2c(n1)ccn2)N(C)C	CHEMBL208957
CCCC[C+]1=NC2(C(=O)N1Cc1ccc(cc1)c1ccccc1c1n[nH]nn1)CCCC2	CHEMBL1513
OCc1ccc(cc1Cl)OC1CC[NH+](CC1)CC1CCN(CC1)c1cccc(n1)C(=O)O	CHEMBL271998
CC(OC[C@@H](C(=O)Nc1ccccn1)Oc1ncnc2c1cnn2c1ncccc1Cl)C	CHEMBL3221499
COc1cnc2c(c1)n(CC[NH+]1CCC(CC1)[NH2+]Cc1cnc(c(c1)C#N)C)c(=O)cn2	CHEMBL3290348
OC(=O)/C=C/c1ccc(cc1)Oc1c(c(=O)oc2c1ccc(c2)O)c1ccc(cc1C)F	CHEMBL3427401
OCc1ccc(cc1Cl)OC1CC[NH+](CC1)CC1CCN(CC1)c1cccc(c1)C(=O)O	CHEMBL270116
COc1cc(ccc1CC(=O)Nc1cn(nc1C)C)Oc1ccnc2c1ccc(c2)OC	CHEMBL2023803
OC(=O)C1CCC(C1)C[NH+]1CCC(CC1)COC(=O)c1c2OCCCn2c2c1cccc2	CHEMBL3329811
O=C(NS(=O)(=O)c1ccc(cc1)OCC[NH+]1CCCCC1)Nc1ccc(cc1)C(F)(F)F	CHEMBL2024393
CCCS(=O)(=O)N1CC[NH+](CC1)C1(CNC(=O)c2c(F)cccc2F)CCC(CC1)(F)F	CHEMBL2324491
C[NH+](CCC(c1ccc(cc1)Cl)NC(=O)C1([NH3+])CCN(CC1)c1ncnc2c1cc[nH]2)C	CHEMBL2325992
N#Cc1ccccc1Cn1c(nc2c(c1=O)cc([nH]2)c1cccnc1)N1CCC[C@H](C1)[NH3+]	CHEMBL2023066
Fc1ccc(cc1)[C@H](C(c1cccnc1)c1cccnc1)c1cccc(n1)NS(=O)(=O)C	CHEMBL4065169
O=C(c1ccc(c(c1)F)S(=O)(=O)Nc1ncns1)NCc1ccc(c(c1)Cl)OC(F)(F)F	CHEMBL4293085
OC[C@@H]1CNc2n(C1)nc(c2c1ccc(=O)n(n1)c1ccccc1C)c1ccc(cc1)F	CHEMBL2170294
O=C(NS(=O)(=O)c1ccc(cc1)OCCC[NH+]1CCCC1)Nc1ccc(cc1)C(F)(F)F	CHEMBL2024399
CNC(=O)c1nccc(c1)Oc1ccc2c(c1)sc(n2)NC(=O)Nc1ccc(c(c1)Cl)Cl	CHEMBL3798983
N#CC1(CC1)NC(=O)[C@@H]1CCCC[C@H]1C(=O)N1CCN(CC1)c1ncc(s1)C(C#N)(C)C	CHEMBL2070948
OC[C@@H](Oc1cc(Oc2ccc(cc2)C(=O)N(C)C)cc(c1)C(=O)Nc1ccn(n1)C)C	CHEMBL3217924
OC[C@@H](Oc1cc(Oc2ccc(cc2)C(=O)N2CCC2)cc(c1)C(=O)Nc1cc[nH]n1)C	CHEMBL3219107
N#Cc1cc(C[NH2+][C@@H]2CC[NH+](C[C@@H]2F)CCn2c(=O)ccc3c2cc(F)cn3)cnc1C	CHEMBL3290350
ONC(=O)CCCCCN(C(=O)c1ccc(cc1)N(C)C)CC(=O)NCc1ccccc1	CHEMBL4069631
CNc1nc2c(n1c1nc3N4CCOC[C@H]4COc3c(n1)C(S(=O)(=O)C)(C)C)cccc2	CHEMBL3985847
O=C(NS(=O)(=O)c1ccc(cc1)OCCC[NH+]1CCCCC1)Nc1ccccc1C(F)(F)F	CHEMBL2021987
CC(OC[C@@H](C(=O)Nc1cnc(cn1)C)Oc1ncnc2c1cnn2c1ccccc1Cl)C	CHEMBL3217794
Fc1cnc2c3c1[C@H](C[NH+]1CCC(CC1)[NH2+]Cc1ncc4c(c1)OCCO4)Cn3c(=O)cc2	CHEMBL2424931
Fc1ccc2c3c1[C@H](C[NH+]1CCC(CC1)[NH2+]Cc1nnc4c(c1)OCCO4)Cn3c(=O)cc2	CHEMBL3793226
CCOC(=O)C1CC[NH+](CC1)CCOc1ccc(cc1)S(=O)(=O)NC(=O)Nc1ccccc1	CHEMBL2024394
O=C(NS(=O)(=O)c1ccc(cc1)OCCC[NH+]1CCCCC1)Nc1ccc(cc1)C(F)(F)F	CHEMBL2021968
OC1C[NH+](C1)C[C@@H](C(=O)Nc1ccccn1)Oc1ncnc2c1cnn2c1ccccc1Cl	CHEMBL3221518
OC(=O)c1cccc(c1)c1cccc(c1O)NN=C1C(=NN(C1=O)c1ccc(c(c1)C)C)C	CHEMBL461101
CC(OC[C@@H](C(=O)Nc1ccc(cn1)C)Oc1ncnc2c1cnn2c1ncccc1Cl)C	CHEMBL3221489
OCc1ccc(cc1Cl)OC1CC[NH+](CC1)CC1CCN(CC1)c1cnc(c(c1)C(=O)O)C	CHEMBL411448

COc1cc2ncnc(c2cc1OC)Oc1ccc(cc1)CC(=O)Nc1cnn(c1)C(C)C	CHEMBL1940274
CCCc1nn(c2c1nc([nH]c2=O)c1cc(ccc1OCC)S(=O)(=O)N1CC[NH+](CC1)C)C	CHEMBL192
O=c1cnc2c3n1[C@H](C[NH+]1CCC(CC1)[NH2+]Cc1ncc4c(c1)CCCO4)Cn3c(=O)cc2	CHEMBL3317856
Fc1ccc(cc1)C[C@H]([NH+]1CCC(CC1)C[NH+]1CCC(CC1)Oc1ccc(cc1)Cl)C(=O)O	CHEMBL2158778
O=C(c1nn(c2c1cccc2)C(C)C)NCC1CC[NH+](CC1)CCc1ccc(cc1)C(=O)O	CHEMBL2179707
N#CC1(CC1)NC(=O)[C@@H]1CCCC[C@H]1C(=O)N1CCN(CC1)c1sc2c(n1)C(C)(C)COC2	CHEMBL2071098
O=C1Nc2c(/C/1=C/c1[nH]ccc1)ccc(c2)NC(=O)c1c[nH]cc(c1=O)c1ccccc1F	CHEMBL3322590
N#Cc1ccc2c(c1)n(CC[NH+]1CC[C@H]([C@H](C1)F)[NH2+]Cc1cnc(c(c1)C#N)C)c(=O)cc2	CHEMBL3290349
CS(=O)(=O)C1(CCOCC1)c1nc(nc2c1OC[C@H]1N2CCOC1)c1cccc2c1cc[nH]2	CHEMBL3921669
COc1ncc2c(n1)n(CC[NH+]1CCC(CC1)[NH2+]Cc1ncc3c(c1)OCCO3)c(=O)cc2	CHEMBL1916543
C[NH+](CCCC(c1ccc(cc1)Cl)NC(=O)C1([NH3+])CCN(CC1)c1ncnc2c1cc[nH]2)C	CHEMBL2325993
CCNC(=O)Nc1ccc(cc1)c1nc2C[NH+](CCc2c(n1)N1CCOC[C@@H]1C)C1COC1	CHEMBL2331680
O=C1COc2c(N1)nc(cc2)C[NH2+]C1CC[NH+](CC1)CCN1C(=O)COc2c1cc(O)cc2	CHEMBL1824030
C[C@H](C(F)(F)F)OC(=O)N1CCC(CC1)O[C@@H]1CC[C@H](CC1)Oc1ncc(nc1)S(=O)(=O)C	CHEMBL3598099
ONC(=O)[C@]1(CCC2(C1)CC[NH2+]CC2)S(=O)(=O)c1ccc(cc1)Oc1ccc(cc1)C(=O)NC	CHEMBL3417770
C[NH+]1CCC(CC1)NC(=O)c1ccc(cc1)Oc1ccc(cc1)C#C[C@]1(O)C[NH+]2CCC1CC2	CHEMBL1940308
N#Cc1cc(cc(c1)c1onc(n1)c1ccc2c(c1)CCN2C(=O)CCC(=O)O)OC(F)(F)F	CHEMBL1916399
O=C(N1CC[NH+](CC1)CC(c1ccc2c(c1C)COC2=O)F)Cc1ccc(nc1)n1cnnn1	CHEMBL3792978
O=C1COc2c(N1)nc(cc2)C[NH2+][C@@H]1CC[C@H](CC1)[C@H](Cn1c(=O)ccc2c1cc(F)cn2)[NH3+]	CHEMBL3355576
Fc1cnc2c3c1[C@](O)(C[NH+]1CCC(CC1)[NH2+]Cc1ncc4c(c1)OCCO4)Cn3c(=O)cc2	CHEMBL2424928
OC1C[NH+](C1)C[C@@H](C(=O)Nc1ccc(cn1)F)Oc1ncnc2c1cnn2c1ncccc1Cl	CHEMBL3221516
N#Cc1ccc2c(c1)n(CC[NH+]1CC[C@H]([C@H](C1)F)[NH2+]Cc1ncc3c(c1)OCCO3)c(=O)cc2	CHEMBL2165065
N#Cc1ccc2c(c1)n(CC[NH+]1CC[C@@H]([C@@H](C1)F)[NH2+]Cc1ncc3c(c1)OCCO3)c(=O)cc2	CHEMBL2165064
O=C(NS(=O)(=O)c1ccc(cc1)OCCCC[NH+]1CCCCC1)Nc1ccc(cc1)C(F)(F)F	CHEMBL2021979
Fc1ccc2c3c1[C@](O)(C[NH+]1CCC(CC1)[NH2+]Cc1ncc4c(c1)OCCO4)Cn3c(=O)cn2	CHEMBL2424933
OC[C@H](OC[C@@H](C(=O)Nc1ccc(cn1)F)Oc1ncnc2c1cnn2c1ncccc1Cl)C	CHEMBL3221513
CO[C@H]1C[NH+](CC[C@H]1[NH2+]Cc1cnc(c(c1)C#N)C)CCn1c(=O)ccc2c1cc(OC)cn2	CHEMBL3290352
COCC[NH+]1CCC[C@@H](C1)Cn1c(nc2c(c1=O)nn(c2)C1CCCCC1)c1cccnc1C	CHEMBL3218892
O=C1COc2c(N1)nc(nc2)C[NH2+][C@@H]1CC[C@H](CC1)[C@H](Cn1c(=O)cnc2c1cc(F)cc2)[NH3+]	CHEMBL3355573
N#Cc1ccc2c(c1)n(CC[NH+]1CC[C@@H]([C@H](C1)F)[NH2+]Cc1ncc3c(c1)OCCO3)c(=O)cc2	CHEMBL2165068
COc1ccc2c(c1)c(ccn2)NC(=O)[C@]1(O)CC[C@H](CC1)[NH2+]Cc1ncc2c(c1)OCCO2	CHEMBL1926866
OCc1ccc(cc1Cl)OC1CC[NH+](CC1)CC1CC[NH+](CC1)[C@H](C(=O)O)Cc1ccccn1	CHEMBL271405
OCc1ccc(cc1Cl)OC1CC[NH+](CC1)CC1CCN(CC1)C(=O)c1ccccc1C(=O)O	CHEMBL272242
OC[C@@H](OC[C@@H](C(=O)Nc1ccc(cn1)Cl)Oc1ncnc2c1cnn2c1ncccc1Cl)C	CHEMBL3221509
OCc1ccc(cc1Cl)OC1CC[NH+](CC1)CC1CC[NH+](CC1)[C@H](C(=O)O)Cc1ccccc1	CHEMBL272842
OC[C@@H](OC[C@@H](C(=O)Nc1ccc(cn1)F)Oc1ncnc2c1cnn2c1ncccc1Cl)C	CHEMBL3221510
COc1ccc2c(c1)n(CC[NH+]1CC[C@H]([C@@H](C1)O)[NH2+]Cc1ncc3c(c1)OCCO3)c(=O)cn2	CHEMBL2164748
Fc1ccc(cc1)C[C@H]([NH+]1CCC(CC1)C[NH+]1CCC(CC1)Oc1ccc(c(c1)C)Cl)C(=O)O	CHEMBL2158782
N#Cc1ccc2c(c1)n(CC[NH+]1CC[C@H]([C@H](C1)O)[NH2+]Cc1ncc3c(c1)OCCO3)c(=O)cc2	CHEMBL2164741
COc1ccc2c(c1)c([C@H](CC[C@@H]1CC[NH+](C[C@@H]1C(=O)O)CCSc1cccs1)O)c(cn2)F	CHEMBL2158050
O=C1COc2c(N1)nc(cc2)C[NH2+][C@H]1CC[NH+](C[C@H]1F)CCn1c(=O)ccc2c1cc(F)cn2	CHEMBL3290354

CO[C@@H]1COCC[C@@H]1[NH2+][C@@H]1CC[C@](C1)(C(C)C)C(=O)N1CCN(CC1)c1scc(n1)C(F)(F)F	CHEMBL1782574
O=C1COc2c(N1)nc(cc2)C[NH2+][C@@H]1CC[C@H](CC1)[C@H](Cn1c(=O)cnc2c1cc(F)cc2)[NH3+]	CHEMBL3355572
N#Cc1cc(cc(c1)c1onc(n1)c1ccc2c(c1)cc1n2CCC1CC(=O)O)OC(F)(F)F	CHEMBL2048293
N#Cc1ccc2c(c1)n(CC[NH+]1CC[C@@H]([C@@H](C1)O)[NH2+]Cc1ncc3c(c1)OCCO3)c(=O)cc2	CHEMBL2164740
OC[C@H](OC[C@@H](C(=O)Nc1ccc(cn1)Cl)Oc1ncnc2c1cnn2c1ncccc1Cl)C	CHEMBL3221512
Fc1ccc(cc1)C[C@H]([NH+]1CCC(CC1)C[NH+]1CCC(CC1)Oc1ccc(cc1C)F)C(=O)O	CHEMBL2158781
ONC(=O)C1(CCC2(C1)CC[NH2+]CC2)S(=O)(=O)c1ccc(cc1)Oc1ccc(cc1)C(=O)NC	CHEMBL3417751
O=C(Nc1ccc(cc1)F)Nc1ccc(cc1)c1nc(cc(n1)N1CCOCC1)CS(=O)(=O)C	CHEMBL2030451
CCOc1ccc(cc1c1nc(=O)c2n([nH]1)c(CCC)nc2C)S(=O)(=O)N1CC[NH+](CC1)CC	CHEMBL1520
ONC(=O)[C@@]1(CCC2(C1)CC[NH2+]CC2)S(=O)(=O)c1ccc(cc1)Oc1ccc(cc1)C(=O)NC	CHEMBL3417769
Clc1ccc(cc1)C1=NN(C[C@@H]1c1ccccc1)[C+](=NS(=O)(=O)c1ccc(cc1)Cl)N=[C+](N)C	CHEMBL4093905
O=C1COc2c(N1)nc(cc2)C[NH2+][C@@H]1CC[NH+](C[C@@H]1F)CCn1c(=O)ccc2c1cc(F)cn2	CHEMBL3290353
C[NH+]1CCC(CC1)Nc1cc(nc2c1cc(Cl)c(c2)OCCC[NH+]1CCCC1)c1ccc(o1)C	CHEMBL4215377
O=C1O[C@@](CCN1[C@H](c1ccc(cc1)c1ccn(c(=O)c1)C)C)(CC(O)(C)C)c1ccccc1	CHEMBL3664717
ONC(=O)C1(CCC2(C1)CC[NH2+]CC2)S(=O)(=O)c1ccc(cc1)OCc1ccc(cc1)C(=O)NC	CHEMBL3417761
O=C(c1ccc(cc1)Oc1ccc(cc1)C#C[C@]1(O)C[NH+]2CCC1CC2)NC1CCCS(=O)(=O)C1	CHEMBL1940315
CCn1nc2c(c1)c(Oc1cnc(c(c1)F)C(=O)N1CCC1)cc(c2)C(=O)Nc1cnc(cn1)C	CHEMBL2204663
O=C1COc2c(N1)nc(nc2)C[NH2+][C@@H]1CC[C@H](CC1)[C@H](Cn1c(=O)cc(c2c1cc(F)cc2)C)[NH3+]	CHEMBL3355569
COc1cnc2c(c1)n(C[C@@H]([C@@H]1CC[C@H](CC1)[NH2+]Cc1ccc3c(n1)NC(=O)CO3)[NH3+])c(=O)cc2	CHEMBL3355575
Fc1ccc(cc1)C[C@]([NH+]1CCC(CC1)C[NH+]1CCC(CC1)Oc1ccc(c(c1)C)Cl)(C(=O)O)C	CHEMBL2158792
COc1ccccc1Oc1ccccc1C[NH+]1CCC2(CC1)CCN(CC2)C(=O)c1ccccn1	CHEMBL271127
Fc1ccc(cc1)C[C@]([NH+]1CCC(CC1)C[NH+]1CCC(CC1)Oc1ccc(cc1C)F)(C(=O)O)C	CHEMBL2158793
Clc1ccc(cc1)C(NC(=O)C1([NH3+])CCN(CC1)c1ncnc2c1cc[nH]2)CCC[NH+]1CCCC1	CHEMBL2325994
OC(=O)CCC(=O)N1CCc2c1ccc(c2)c1noc(n1)c1cc(cc(c1)C(F)(F)F)C(F)(F)F	CHEMBL1916559
OCc1ccc(cc1Cl)OC1CC[NH+](CC1)CC1CCN(CC1)C(=O)c1cc(C)ccc1C(=O)O	CHEMBL429763
CS(=O)(=O)N1CC[NH+](CC1)Cc1cn2c(n1)c(nc(c2)c1cccc2c1cn[nH]2)N1CCOCC1	CHEMBL1957497
CO[C@H]1C[NH+](CC[C@@H]1[NH2+]Cc1ncc2c(c1)OCCO2)CCn1c(=O)ccc2c1cc(C#N)cc2	CHEMBL2165058
N#Cc1ccc2c(c1)n(CC[NH+]1CC[C@H]([C@H](C1)O)[NH2+]Cc1ccc3c(n1)NC(=O)CO3)c(=O)cc2	CHEMBL2164743
COc1cnc2c(c1)n(CC[NH+]1CC[C@@H]([C@@H](C1)F)[NH2+]Cc1ccc3c(n1)NC(=O)CO3)c(=O)cc2	CHEMBL3290356
COc1cc(F)c2c(c1)c(ccn2)NC(=O)[C@]1(O)CC[C@H](CC1)[NH2+]Cc1ncc2c(c1)OCCO2	CHEMBL1926865
N#CC1(CC1)NC(=O)[C@@H]1CCCC[C@H]1C(=O)N1CCN(CC1)c1nc2c(s1)cc(cc2)S(=O)(=O)C	CHEMBL2070947
COc1cc2c(NC3CC[NH+](CC3)C)cc(nc2cc1OC1CC2(C1)C[NH+](C2)C)c1ccc(o1)C	CHEMBL4215326
Fc1cnc2c3c1[C@](O)(C[NH+]1CCC(CC1)[NH2+]Cc1ncc4c(c1)OCCO4)Cn3c(=O)c(c2)F	CHEMBL2424934
COC(c1ccc2c(c1C)COC2=O)C[NH+]1CCN(CC1)C(=O)Cc1ccc(nc1)n1cnnn1	CHEMBL3792808
COc1ccc2c(c1)n(CC[NH+]1CC[C@H]([C@H](C1)O)[NH2+]Cc1ccc3c(n1)NC(=O)CO3)c(=O)cn2	CHEMBL2164747
CO[C@H]1C[NH+](CC[C@H]1[NH2+]Cc1ncc2c(c1)OCCO2)CCn1c(=O)ccc2c1cc(C#N)cc2	CHEMBL2165054
N#Cc1ccc2c(c1)n(CC[NH+]1CC[C@@H]([C@@H](C1)O)[NH2+]Cc1ccc3c(n1)NC(=O)CO3)c(=O)cc2	CHEMBL2164742
N#Cc1ccc2c(c1)n(CC[NH+]1CC[C@H]([C@H](C1)F)[NH2+]Cc1ccc3c(n1)NC(=O)CO3)c(=O)cc2	CHEMBL2165066
COc1cnc2c(c1)n(CC[NH+]1CC[C@H]([C@H](C1)F)[NH2+]Cc1ccc3c(n1)NC(=O)CO3)c(=O)cc2	CHEMBL3290355
COc1ccc2c(c1)n(CC[NH+]1CC[C@@H]([C@@H](C1)O)[NH2+]Cc1ccc3c(n1)NC(=O)CO3)c(=O)cn2	CHEMBL2164746
COC[C@@H](Oc1cc(Oc2ncc(nc2)C(=O)N2CCC2)cc(c1)C(=O)Nc1ncc(nc1)C)C	CHEMBL3219124

COc1ccc2c(c1)c(/C=N/NS(=O)(=O)c1ccc(cc1)C)cn2CC(=O)Nc1ccc(cc1)Cl	CHEMBL2440387
COc1ccc2c(c1)c([C@H](CC[C@@H]1CC[NH+](C[C@@H]1C(=O)O)C1CC(C1)c1ccno1)O)c(cn2)F	CHEMBL4101570
CCOC(=O)C1CC[NH+](CC1)CCCCOc1ccc(cc1)S(=O)(=O)NC(=O)Nc1ccccc1	CHEMBL2021980
COc1ccc2c(c1)n(C[C@@H]([C@@H]1CC[C@H](CC1)[NH2+]Cc1ncc3c(n1)NC(=O)CO3)[NH3+])c(=O)cn2	CHEMBL3352849
O=C1COc2c(N1)nc(cc2)C[NH2+]C1CC[NH+](CC1)CCN1C(=O)COc2c1cc(cc2)C(O)C	CHEMBL1824029
COc1cc2c(NC3CC[NH+](CC3)C)cc(nc2cc1OCCC[NH+]1CCCC1)c1ccc(o1)C	CHEMBL4170114
COc1cnc2c(c1)n(C[C@H]([C@@H]1CC[C@H](CC1)[NH2+]Cc1ccc3c(n1)NC(=O)CO3)[NH3+])c(=O)cc2	CHEMBL3355578
O=C1COc2c(N1)nc(cc2)C[NH2+][C@@H]1CC[C@H](CC1)[C@H](Cn1c(=O)cc(c2c1cc(F)cc2)C)[NH3+]	CHEMBL3355570
O=C1COc2c(N1)nc(cc2)C[NH2+]C1CC[NH+](CC1)CCN1C(=O)COc2c1cc(cc2)C(=O)O	CHEMBL1824033
COc1ccc2c(c1)n(C[C@@H]([C@@H]1CC[C@H](CC1)[NH2+]Cc1ncc3c(n1)NC(=O)CO3)[NH3+])c(=O)cc2	CHEMBL3355568
CC(=O)N1CCN(CC1)c1cnc2c(c1)c(NCc1cccc(c1)[N+](=O)[O-])cc(c2)C(F)(F)F	CHEMBL3221132
ONC(=O)C1(CCC2(C1)CC[NH2+]CC2)S(=O)(=O)c1ccc(cc1)Oc1ccc(cc1)OC(F)(F)F	CHEMBL3417745
COc1ccc2c(c1)n(C[C@@H]([C@@H]1CC[C@H](CC1)[NH2+]Cc1ccc3c(n1)NC(=O)CO3)[NH3+])c(=O)cn2	CHEMBL3355571
CNC(=O)c1nccc(c1)Oc1ccc2c(c1)sc(n2)NC(=O)Nc1ccc(c(c1)C(F)(F)F)Cl	CHEMBL3799746
C1C[NH2+]CC[NH+](CCC[NH2+]CC[NH2+]C1)Cc1ccc(cc1)C[NH+]1CC[NH2+]CCC[NH2+]CC[NH2+]CCC1	CHEMBL2311028
CO[C@@H]1C[NH+](CC[C@H]1[NH2+]Cc1ccc2c(n1)NC(=O)CO2)CCn1c(=O)ccc2c1cc(C#N)cc2	CHEMBL2165059
OC[C@H](OC[C@@H](C(=O)Nc1ncc(nc1)C)Oc1ncnc2c1cnn2c1c(Cl)cccc1C#N)C	CHEMBL3221532
O=C(NS(=O)(=O)c1ccc(cc1)OCCC[NH+]1CCCCC1)NC(c1ccccc1)c1ccccc1	CHEMBL2021992
COc1cc2c(cc1F)ncc(=O)n2C[C@@H]([C@@H]1CC[C@H](CC1)[NH2+]Cc1ncc2c(n1)NC(=O)CO2)[NH3+]	CHEMBL3355574
COc1ccccc1Oc1ccccc1C[NH+]1CCC2(CC1)CCN(CC2)C(=O)c1nccc(c1)N	CHEMBL272846
COc1ccccc1Oc1ccccc1C[NH+]1CCC2(CC1)CCN(CC2)C(=O)c1ccnc(c1)N	CHEMBL429924
OC(=O)/C=C/c1ccc(cc1)Oc1c2ccc(cc2oc(=O)c1c1ccc(cc1C)OC(F)(F)F)O	CHEMBL3427402
CO[C@H]1C[NH+](CC[C@H]1[NH2+]Cc1ccc2c(n1)NC(=O)CO2)CCn1c(=O)ccc2c1cc(OC)cn2	CHEMBL3290357
CO[C@H]1C[NH+](CC[C@H]1[NH2+]Cc1ccc2c(n1)NC(=O)CO2)CCn1c(=O)ccc2c1cc(C#N)cc2	CHEMBL2165055
Fc1cnc2c3c1[C@@](O)(Cn3c(=O)cc2)CC12CCC(CC1)(CO2)[NH2+]Cc1ncc2c(c1)OCCO2	CHEMBL3425810
COc1cc2c(NC3CCN(C(=O)C3)C)cc(nc2cc1OCCC[NH+]1CCCC1)c1ccc(o1)C	CHEMBL4217332
O=C1COc2c(N1)nc(cc2)C[NH2+]C12CCC(CC1)(OC2)C[C@@]1(O)Cn2c3c1c(F)cnc3ccc2=O	CHEMBL3400818
OC(=O)C(c1ccc(cc1)C(CCC[NH+]1CCC(CC1)C(c1ccccc1)(c1ccccc1)O)O)(C)C	CHEMBL914
CCS(=O)(=O)c1ccc2c(c1)N(CC[NH+]1CCC(CC1)[NH2+]Cc1ccc3c(n1)NC(=O)CO3)C(=O)CO2	CHEMBL1824038
O=C1COc2c(N1)nc(cc2)C[NH2+]C12CCC(CC1)(OC2)C[C@]1(O)Cn2c3c1c(F)cnc3ccc2=O	CHEMBL3400817
COc1cc2c(NC3CC4CCC(C3)[NH+]4C)cc(nc2cc1OCCC[NH+]1CCCC1)c1ccc(o1)C	CHEMBL4205373
N#CC1=C[C@@]2(C)[C@H](C(C1=O)(C)C)CC[C@@]1(C2=CC(=O)[C@H]2[C@@]1(C)CC[C@@]1([C@H]2CC(C)(C)C C1)C(=O)OC)C	CHEMBL1762621
CC(=O)N1CC[NH+](CC1)CCOc1ccc(cc1)C1CCN(CC1)[C+]1=Nn2c(CC1)nnc2C(F)(F)F	CHEMBL2346976
O=C1COc2c(N1)nc(cc2)C[NH2+]C1CC[NH+](CC1)CCN1C(=O)COc2c1cc(cc2)n1cnnn1	CHEMBL1824028
ONC(=O)C1(CCC2(C1)CC[NH2+]CC2)S(=O)(=O)c1ccc(cc1)Oc1ccc(cc1)C(=O)N1CCCC1	CHEMBL3417752
O=C(C1CCC(CC1)(F)F)N[C@H](c1ccccc1)CC[NH+]1[C@@H]2CC[C@H]1C[C@@H](C2)n1c(C)nnc1C(C)C	CHEMBL1201187
ONC(=O)C1(CN(C1)C(=O)C(C)C)N(S(=O)(=O)c1ccc(cc1)OCc1cc(C)nc2c1cccc2)C	CHEMBL4215407
COc1cc2c(NCC3CC[NH+](CC3)C)cc(nc2cc1OCCC[NH+]1CCCC1)c1ccc(o1)CC	CHEMBL4212905
O=C1COc2c(N1)nc(c(c2)F)C[NH2+]C12CCC(CC1)(OC2)C[C@]1(O)Cn2c3c1c(F)cnc3ccc2=O	CHEMBL3425799
OCc1ccc(cc1Cl)OC1CC[NH+](CC1)CC1CC[NH+](CC1)C(C(=O)O)Cc1ccc(cc1)S(=O)(=O)C	CHEMBL429581

COc1ccccc1Oc1ccccc1C[NH+]1CCC2(CC1)CCN(CC2)C(=O)c1ccccc1C(=O)O	CHEMBL272441
CC(CN(S(=O)(=O)c1ccc(cc1)N)C[C@H]([C@H](Cc1ccccc1)NC(=O)O[C@H]1CO[C@@H]2[C@H]1CCO2)O)C	CHEMBL1323
CNC(=O)c1nccc(c1)Oc1ccc2c(c1)sc(n2)NC(=O)Nc1cc(cc(c1)C(F)(F)F)C(F)(F)F	CHEMBL3797601
COc1cc2c(NC3CCC43CC[NH+](CC4)C)cc(nc2cc1OCCC[NH+]1CCCC1)c1ccc(o1)C	CHEMBL4208812
O=C1COc2c(N1)nc(cc2)C[NH2+]C1CC[NH+](CC1)CCn1c(=O)ccc2c1nc(cc2)Oc1nocc1	CHEMBL3608747
O=C1COc2c(N1)nc(c(c2)Cl)C[NH2+]C12CCC(CC1)(OC2)C[C@]1(O)Cn2c3c1c(F)cnc3ccc2=O	CHEMBL3425800
O=C1COc2c(N1)nc(cc2)C[NH2+]C1CC[NH+](CC1)CCn1c(=O)ccc2c1cc(cn2)O[C@@H]1COCC1	CHEMBL3608752
COc1cc2c(NC3CC4(C3)CC[NH+](CC4)C)cc(nc2cc1OCCC[NH+]1CCCC1)c1ccc(o1)C	CHEMBL4206035
CC(=O)N1CC[NH+](CC1)CCOc1ccc(cc1)C1(O)CCN(CC1)c1ccc2n(n1)c(nn2)C(F)(F)F	CHEMBL2346975
O=C1COc2c(N1)nc(cc2)C[NH2+]C1CC[NH+](CC1)CCn1c(=O)ccc2c1cc(cn2)O[C@H]1COCC1	CHEMBL3608751
ONC(=O)C1(CN(C1)C(=O)CC(C)C)N(S(=O)(=O)c1ccc(cc1)OCc1cc(C)nc2c1cccc2)C	CHEMBL4208519
O=C1COc2c(N1)nc(cc2)C[NH2+]C1CC[NH+](CC1)CCn1c(=O)ccc2c1cc(cn2)Oc1nocc1	CHEMBL3608748
Fc1ccc2c(c1)oc(cc2=O)C(=O)NC1CC[NH+](CC1)Cc1ccc(c(c1)F)C(=O)NCC[NH+]1CCCC1	CHEMBL245846
O=C1COc2c(N1)nc(cc2)C[NH2+]C1CC[NH+](CC1)CCN1C(=O)COc2c1cc(cc2)NC(=O)C(F)(F)F	CHEMBL1824032
OC[C@H]1CCC[NH+]1CCCOc1ccc2c(c1)cnnc2Nc1cnn(c1)CC(=O)Nc1cccc(c1F)F	CHEMBL253968
O=C1COc2c(N1)nc(cc2)C[NH2+][C@@H]1CC[NH+](C[C@@H]1F)CCn1c(=O)ccc2c1cc(cn2)Oc1nocc1	CHEMBL3608756
O=C1COc2c(N1)nc(cc2)C[NH2+]C1CC[NH+](CC1)CCn1c(=O)ccc2c1cc(cn2)Oc1cccnc1	CHEMBL3608749
O=C1COc2c(N1)nc(cc2)C[NH2+]C1CC[NH+](CC1)CCn1c(=O)ccc2c1nc(cc2)Oc1cncnc1	CHEMBL3608746
CC([C@H](C(=O)O)Oc1ccc(cc1)CNC(=O)[C@@H]1CCCN1C(=O)CC(Cc1c(F)c(F)c(c(c1F)F)F)[NH3+])C	CHEMBL183289
O[C@@H]([C@@H](NC(=O)c1cccc(c1C)O)CSc1ccccc1)C[NH+]1C[C@H]2CCCC[C@H]2C[C@H]1C(=O)NC(C)(C)C	CHEMBL584
O=C1COc2c(N1)nc(cc2)C[NH2+]C1CC[NH+](CC1)CCn1c(=O)ccc2c1cc(OCc1ccn(n1)C)cn2	CHEMBL3608755
Fc1cccc(c1)COc1ccc(cc1Cl)Nc1ncnc2c1cc(cc2)c1ccc(o1)C[NH2+]CCS(=O)(=O)C	CHEMBL554
COc1ccccc1Oc1ccccc1C[NH+]1CCC2(CC1)CCN(CC2)C(=O)c1cc(Cl)c(cc1C(=O)O)Cl	CHEMBL408483
COc1ccccc1Oc1ccccc1C[NH+]1CCC2(CC1)CCN(CC2)C(=O)c1ccccc1c1[nH]nnn1	CHEMBL410862
O=C1COc2c(N1)nc(cc2)C[NH2+]C1CC[NH+](CC1)CCn1c(=O)ccc2c1cc(OCc1ccccn1)cn2	CHEMBL3608753
O=C1COc2c(N1)nc(cc2)C[NH2+]C1CC[NH+](CC1)CCn1c(=O)ccc2c1cc(OCc1cccnn1)cn2	CHEMBL3608754
N#Cc1cc(C[NH2+]C2CC[NH+](CC2)CCn2c(=O)ccc3c2cc(cn3)Oc2ccc(cc2)S(=O)(=O)N)cnc1C	CHEMBL3608745
O=C1CCc2c(N1)nccc2Oc1ccc2c(c1)C[C@@H](CC2)NC(=O)c1cc(cc(c1)C(F)(F)F)c1ncn(c1)C	CHEMBL3763694
O=C1CCc2c(N1)nccc2Oc1ccc2c(c1)C[C@@H](CC2)NC(=O)c1cc(cc(c1)C(F)(F)F)c1cnn(c1)C	CHEMBL3763646
O=C(Nc1cccc(c1F)F)Cn1ncc(c1)Nc1nncc2c1ccc(c2)OCCC[NH+]1CCC[C@@H]1COP(=O)(O)O	CHEMBL250766
OC(=O)c1ccccc1C(=O)N1CCC(CC1)(CC[NH+]1C2CCC1CC(C2)n1c(C)nc2c1cccc2)c1ccccc1	CHEMBL1784408
OC(=O)CC1CCC(CC1)c1ccc(cc1)c1[nH]c2c([nH+]1)cc(cc2)NC(=O)c1nc(oc1C(F)(F)F)c1ccccc1	CHEMBL2409564
O=C1COc2c(N1)nc(cc2)C[NH2+]C1CC[NH+](CC1)CCn1c(=O)ccc2c1nc(cc2)Oc1ccc(cc1)S(=O)(=O)N	CHEMBL3608743
OCC1COCC[NH+]1Cc1cc(cc(c1)C(F)(F)F)C(=O)N[C@@H]1CCc2c(C1)cc(cc2)Oc1ccnc2c1CCC(=O)N2	CHEMBL3764275
COc1c(cc(cc1OC(C)(C)C)CN1CC(=O)N(CC1=O)CCCCCC(c1ccc(cc1)F)c1ccc(cc1)F)OC(C)(C)C	CHEMBL3356240
Fc1cccc(c1)C1(CCN(CC1)C(=O)c1cc(NS(=O)(=O)C)c(cc1F)F)CC[NH+]1C2CCC1CC(C2)n1c(C)nc2c1cccc2	CHEMBL1784523
CC[C@H]1OC(=O)[C@H](C)[C@@H](O[C@@H]2O[C@@H](C)[C@@H]([C@](C2)(C)OC)O)[C@H](C)[C@@H](O[C@@H]2O[C@H](C)C[C@@H]([C@H]2O)[NH+](C)C)[C@](C[C@H]([C@@H]([C@@H]([C@H]([C@]1(C)O)O)C)[NH3+])C)(C)O	CHEMBL290242
N#CC1=C(OCc2ccc(cc2)NC(=O)CC[C@@H](C(=O)OC)[NH3+])C(C)(C)[C@H]2[C@@]([C@H]1O)(C)C1=CC(=O)[C@H]3[C@@]([C@@]1(CC2)C)(C)CC[C@@]1([C@H]3CC(C)(C)CC1)C(=O)OC	CHEMBL4083511

CC[C@H]1OC(=O)[C@H](C)C(=O)[C@H](C)[C@@H](O[C@@H]2O[C@H](C)C[C@@H]([C@H]2O)[NH+](C)C)[C@](C[C@H](C(=O)[C@@H]([C@@H]2[C@]1(C)OC(=O)N2C1C[NH+](C1)Cc1ccnc2c1nccc2)C)C)(C)O	CHEMBL583090
CC[C@H]1OC(=O)[C@H](C)C(=O)[C@H](C)[C@@H](O[C@@H]2O[C@H](C)C[C@@H]([C@H]2O)[NH+](C)C)[C@](C[C@H](C(=O)[C@@H]([C@@H]2[C@]1(C)OC(=O)N2C1C[NH+](C1)Cc1c(O)cnc2c1cccn2)C)C)(C)O	CHEMBL583298
O=CC[C@H]1C[C@@H](C)[C@@H](O)C=CC=CC[C@H](OC(=O)C[C@H]([C@@H]([C@H]1O[C@@H]1O[C@H](C)[C@H]([C@@H]([C@H]1O)[NH+](C)C)O[C@@H]1O[C@@H](C)[C@@H]([C@](C1)(C)O)OC(=O)CC(C)C)OC)OC(=O)C)C	CHEMBL1671903
CNC(=O)CO[C@H]1[C@H](C)C[C@@](C)(O)[C@H](O[C@@H]2O[C@H](C)C[C@@H]([C@H]2O)[NH+](C(C)C)C)[C@@H](C)[C@H](O[C@@H]2O[C@@H](C)[C@@H]([C@](C2)(C)OC)O)[C@H](C(=O)O[C@@H]([C@@]([C@@H]([C@H]1C)O)(C)O)CC)C	CHEMBL572996
O[C@@H]1CC[C@@H](O)[C@H](O)C[C@H](O)C[C@]2(O)C[C@H](O)[C@H]([C@@H](O2)C[C@H](/C=C/C=C/C=C/C=C/C=C/C=C/C=C/[C@@H]([C@H]([C@H]([C@@H](OC(=O)C[C@@H](C1)O)C)C)O)C)O[C@@H]1O[C@H](C)[C@H]([C@@H]([C@@H]1O)[NH3+])O)C(=O)O	CHEMBL267345
OCCO[C@@H]1CC[C@H](C[C@H]1OC)C[C@H]([C@H]1OC(=O)[C@@H]2CCCCN2C(=O)C(=O)[C@]2(O)O[C@@H](CC[C@H]2C)C[C@H](OC)/C(=C/C=C/C=C/[C@H](C[C@H](C(=O)[C@@H]([C@@H](C(=C[C@H](C(=O)C1)C)C)O)OC)C)C)/C)C	CHEMBL1908360
CC[C@@H]([C@H](C(=O)N[C@H](C(=O)N[C@H](C(=O)N[C@@H]1[C@H](C)OC(=O)[C@@H](NC(=O)[C@@H](NC(=O)[C@@H](NC1=O)C)C[C@H]1CN[C+](=[NH2+])N1)[C@H](CC)C)CO)[C@H](CC)C)NC(=O)[C@H](NC(=O)[C@@H](NC(=O)[C@H]([C@H](CC)C)NC(=O)[C@@H](Cc1ccccc1)[NH2+]C)CO)CCC(=O)N)C	CHEMBL3977597

Table S3. Modulators of Kv10.1 that do not bind into the central cavity that were used as the validation set for the final merged pharmacophore model.

**SMILES**	**Name**
C[NH+](C)CCCOc1c(Br)cc(CCNC(=O)c2ccccc2Br)cc1Br	Purp1 [8]
COc1ccc(C(=O)NCCc2cc(Br)c(OCCC[NH+](C)C)c(Br)c2)cc1	Purp2 [8]
C[NH2+]CCCOc1c(Br)cc(CCNC(=O)c2ccc(OC)cc2)cc1Br	Purp3 [8]
C[NH+](C)CCCOc1c(Br)cc(CCNC(=O)c2cc(C(F)(F)F)cc(C(F)(F)F)c2)cc1Br	Purp4 [8]
C[NH+](C)CCCOc1c(Br)cc(CCNC(=O)c2ccccc2F)cc1Br	Purp5 [8]
C[NH+](C)CCCOc1c(Br)cc(CCNC(=O)c2cccc(Br)c2)cc1Br	Purp6 [8]
C[NH+](C)CCCOc1c(Br)cc(CCNC(=O)c2ccc(Br)cc2)cc1Br	Purp7 [8]
Cc1cc(C(=O)NCCc2cc(Br)c(OCCC[NH+](C)C)c(Br)c2)no1	Purp8 [8]
COc1ccc(C(=O)NCCc2cc(Br)c(OCCC[NH+](C)C)c(Br)c2)cc1Cl	Purp9 [8]
C[NH+](C)CCCOc1c(Br)cc(CCNC(=O)c2cc(Cl)cc(Cl)c2)cc1Br	Purp10 [8]
CC(C)CCCOc1c(Br)cc(CCNC(=O)c2cc(Cl)cc(Cl)c2)cc1Br	Purp11 [8]
C[NH+](C)CCCOc1c(Br)cc(CCNC(=O)c2cncc(Br)c2)cc1Br	Purp12 [8]
CC(C)CCCOc1c(Br)cc(CCNC(=O)c2cc(Br)cc(Br)c2)cc1Br	Purp13 [8]
C[NH2+]CCCOc1c(Br)cc(CCNC(=O)c2cc(Cl)c(OC)c(Cl)c2)cc1Br	Purp14 [8]
CCOc1c(Cl)cc(C(=O)NCCc2cc(Br)c(OCCC[NH+](C)C)c(Br)c2)cc1Cl	Purp15 [8]
COc1c(Cl)cc(C(=O)NCCc2cc(Br)c(OCCC[NH+](C)C)c(Br)c2)cc1Cl	Purp16 [8]
C[NH2+]CCCOc1c(Br)cc(CCNC(=O)c2ccc(OC)c(Cl)c2)cc1Br	Purp17 [8]
C[NH+](C)CCCOc1ccc(CCNC(=O)c2cc(Cl)cc(Cl)c2)cc1	Purp18 [8]
C[NH+](C)CCCOc1c(Br)cc(CCNC(=O)c2cc(F)cc(F)c2)cc1Br	Purp19 [8]
COc1ccc(C(=O)NCCc2cc(Br)c(OCCC[NH+](C)C)c(Br)c2)cc1Br	Purp20 [8]
COc1c(Br)cc(C(=O)NCCc2cc(Br)c(OCCC[NH+](C)C)c(Br)c2)cc1Br	Purp21 [8]
C[NH2+]CCCOc1c(Br)cc(CCNC(=O)c2cc(F)c(OC)c(F)c2)cc1Br	Purp22 [8]
COc1ccc(C(=O)NCCc2cc(Br)c(OCCC[NH+](C)C)c(Br)c2)cc1I	Purp23 [8]
COc1c(F)cc(C(=O)NCCc2cc(Br)c(OCCC[NH+](C)C)c(Br)c2)cc1F	Purp24 [8]
COc1ccc(C(=O)NCCc2cc(Br)c(OCCC[NH+](C)C)c(Br)c2)cc1F	Purp25 [8]
COc1ccc(C(=O)NCCc2cc(Br)c(OCCC[NH+](C)C)c(Br)c2)cc1C	Purp26 [8]
COc1ccc(C(=O)NCCc2cc(Br)c(OCCC[NH+](C)C)c(Br)c2)cc1OC	Purp27 [8]
CCCCc1oc2ccccc2c1C(=O)c1cc(I)c(OCC[NH+](CC)CC)c(I)c1	Amiodarone [5]
COCC(=O)O[C@]1(CC[NH+](C)CCCc2nc3ccccc3[nH]2)CCc2cc(F)ccc2[C@@H]1C(C)C	Mibefradil [5]
O=C(Nc1ccc(Oc2ccccc2)cc1)c1cccc([N+](=O)[O-])c1	ICA105574 [5]
CC(=CCCC(C)(O)C1CCC2(C)C1C(O)CC1C3(C)CCC(OC4OC(CO)C(O)C(O)C4OC4OC(CO)C(O)C(O)C4O)C(C)(C)C3CCC2 1C)C	Ginsenoside [5]
COc1ccc2cc1Oc1ccc(cc1)C[C@H]1c3cc(c(OC)cc3CC[NH+]1C)Oc1c(OC)c(OC)cc3c1[C@H](C2)[NH+](C)CC3	Tetrandrine [5]
Oc1cc(O)c2c(c1)O[C@H](c1ccc(O)c(O)c1)[C@H](O)[C@H]2c1c(O)cc(O)c2c1O[C@H](c1ccc(O)c(O)c1)[C@@H](O)C2	ProcyanidinB1 [5]
C[NH+](C)CCCN1c2ccccc2Sc2ccc(Cl)cc12	Chlorpromazine [5]

C[NH+](C)CCCOc1ccc(Nc2ccc(C(F)(F)F)cc2[N+](=O)[O-])cc1	1 [9]
C[NH+](C)CCCOc1ccc(Nc2ccccc2[N+](=O)[O-])cc1	4 [9]
C[NH+](C)CC(O)COc1ccc(Nc2ccccc2[N+](=O)[O-])cc1	7 [9]
C[NH+](C)CC(O)COc1ccc(Nc2ccc(C(F)(F)F)cc2[N+](=O)[O-])cc1	ZVS-08 [9]
CC(=O)NCC(O)COc1ccc(Nc2ccc(C(F)(F)F)cc2[N+](=O)[O-])cc1	12 [9]
COC(=O)c1ccccc1Nc1ccc(OCC(O)C[NH+](C)C)cc1	9 [9]
C[NH+](C)CC(O)COc1ccc(Nc2ccccc2C(=O)[O-])cc1	11 [9]
C[NH+](C)CC(O)COc1ccc(Nc2ccccc2C(=O)[O-])cc1	2 [9]
O=[N+]([O-])c1cc(C(F)(F)F)ccc1Nc1ccc(OCC(O)CNc2ccccc2)cc1	5 [9]
O=[N+]([O-])c1cc(C(F)(F)F)ccc1Nc1ccc(OCC(O)C[NH+]2CCOCC2)cc1	8 [9]
C[NH+](C)CC(O)COc1ccc(Nc2ccc(C(F)(F)F)cc2N)cc1	10 [9]
C[NH+](C)CC(O)COc1ccc(Nc2ccccc2)cc1	3 [9]
C[NH+](C)CC(O)COc1ccc(Nc2ccc(C(F)(F)F)cc2)cc1	6 [9]

References

1. 1. Whicher, J.R.; MacKinnon, R. Structure of the Voltage-Gated K+ Channel Eag1 Reveals an Alternative Voltage Sensing Mechanism. *Science* **2016**, *353*, 664–669.
2. 2. Wang, W.; MacKinnon, R. Cryo-EM Structure of the Open Human Ether-à-Go-Go -Related K + Channel HERG. *Cell* **2017**, *169*, 422-430.e10.
3. 3. Smart, O.S.; Goodfellow, J.M.; Wallace, B.A. The Pore Dimensions of Gramicidin A. *Biophys. J.* **1993**, *65*, 2455–2460.
4. 4. Friesner, R.A.; Murphy, R.B.; Repasky, M.P.; Frye, L.L.; Greenwood, J.R.; Halgren, T.A.; Sanschagrin, P.C.; Mainz, D.T. Extra Precision Glide: Docking and Scoring Incorporating a Model of Hydrophobic Enclosure for Protein−Ligand Complexes. *J. Med. Chem.* **2006**, *49*, 6177–6196.
5. 5. Toplak, Ž.; Hendrickx, L.A.; Abdelaziz, R.; Shi, X.; Peigneur, S.; Tomašič, T.; Tytgat, J.; Peterlin-Mašič, L.; Pardo, L.A. Overcoming Challenges of HERG Potassium Channel Liability through Rational Design: Eag1 Inhibitors for Cancer Treatment. *Med. Res. Rev.* **2021**, med.21808.
6. 6. Mendez, D.; Gaulton, A.; Bento, A.P.; Chambers, J.; De Veij, M.; Félix, E.; Magariños, M.P.; Mosquera, J.F.; Mutowo, P.; Nowotka, M.; et al. ChEMBL: Towards Direct Deposition of Bioassay Data. *Nucleic Acids Res.* **2019**, *47*, D930–D940.
7. 7. Berthold, M.R.; Cebron, N.; Dill, F.; Gabriel, T.R.; Kötter, T.; Meinl, T.; Ohl, P.; Sieb, C.; Thiel, K.; Wiswedel, B. KNIME: The Konstanz Information Miner. In Proceedings of the Studies in Classification, Data Analysis, and Knowledge Organization (GfKL 2007); Springer, 2007.
8. 8. Moreels, L.; Bhat, C.; Voráčová, M.; Peigneur, S.; Goovaerts, H.; Mäki-Lohiluoma, E.; Zahed, F.; Pardo, L.A.; Yli-Kauhaluoma, J.; Kiuru, P.; et al. Synthesis of Novel Purpurealidin Analogs and Evaluation of Their Effect on the Cancer-Relevant Potassium Channel Kv10.1. *PLoS One* **2017**, *12*, e0188811.
9. 9. Toplak, Ž.; Hendrickx, L.A.; Gubič, Š.; Možina, Š.; Žegura, B.; Štern, A.; Novak, M.; Shi, X.; Peigneur, S.; Tytgat, J.; et al. 3D Pharmacophore-Based Discovery of Novel Kv10.1 Inhibitors with Antiproliferative Activity. *Cancers* **2021**, *13*, 1244.
10.
